# Immune System and Neuroinflammation in Idiopathic Parkinson’s Disease: Association Analysis of Genetic Variants and miRNAs Interactions

**DOI:** 10.3389/fgene.2021.651971

**Published:** 2021-06-03

**Authors:** Claudia Strafella, Valerio Caputo, Andrea Termine, Francesca Assogna, Clelia Pellicano, Francesco E. Pontieri, Lucia Macchiusi, Giulietta Minozzi, Stefano Gambardella, Diego Centonze, Paola Bossù, Gianfranco Spalletta, Carlo Caltagirone, Emiliano Giardina, Raffaella Cascella

**Affiliations:** ^1^Genomic Medicine Laboratory, IRCCS Santa Lucia Foundation, Rome, Italy; ^2^Medical Genetics Laboratory, Department of Biomedicine and Prevention, Tor Vergata University, Rome, Italy; ^3^Laboratory of Neuropsychiatry, Department of Clinical and Behavioral Neurology, IRCCS Santa Lucia Foundation, Rome, Italy; ^4^Department of Neuroscience, Mental Health and Sensory Organs, “Sapienza” Università di Roma, Rome, Italy; ^5^Department of Veterinary Medicine, University of Milan, Milan, Italy; ^6^Neuromed Institute IRCCS, Pozzilli, Italy; ^7^Department of Biomolecular Sciences, University of Urbino “Carlo Bo”, Urbino, Italy; ^8^Laboratory of Experimental Neuropsychobiology, Department of Clinical and Behavioral Neurology, IRCCS Santa Lucia Foundation, Rome, Italy; ^9^Department of Clinical and Behavioral Neurology, IRCCS Fondazione Santa Lucia, Rome, Italy; ^10^Department of Biomedical Sciences, Catholic University Our Lady of Good Counsel, Tirana, Albania

**Keywords:** Parkinson’s disease, genetics, susceptibility, *IL6*, therapeutic target, miRNA, neuroinflammation

## Abstract

The present study investigated the association of SNPs involved in the regulation of immune response, cellular degenerative and neuroinflammatory pathways with the susceptibility and progression of idiopathic Parkinson’s Disease (PD). In particular, 342 PD patients were subjected to a genotyping analysis of a panel of 120 SNPs by Open Array Technology. As control group, 503 samples representative of the European general population were utilized. The genetic analysis identified 26 SNPs associated with PD susceptibility. Of them, 12 SNPs were described as significant expression Quantitative Loci (eQTL) variants in different brain regions associated with motor and non-motor PD phenomenology. Moreover, the study highlighted 11 novel susceptibility genes for PD, which may alter multiple signaling pathways critically involved in peripheral immune response, neuroinflammation, neurodegeneration and dopaminergic neurons wiring. The study of miRNA-target genes highlighted a possible role of miR-499a, miR-196a2, and miR-29a in the modulation of multiple neuroinflammatory and neurodegenerative mechanisms underlying PD physiopathology. The study described a network of interconnected genes (*APOE*, *CLU*, *IL6*, *IL7R*, *IL12B*, *INPP5D*, *MAPK1*, *MEF2C*, *MIF*, and *TNFSF14*), which may act as upstream regulators in the modulation of biological pathways relevant to PD. Intriguingly, *IL6* stands out as a master gene regulator since it may indirectly regulate the network of interconnected genes. The study highlighted different genes and miRNAs interactions potentially involved in PD physiopathology, which are worth to be further explored to improve the knowledge of disease and the research of novel treatments strategies.

## Introduction

The functional analysis of the human genome enhanced the understanding of the genetic basis of Parkinson’s Disease (PD), which accounts for both common and rare genetic variants associated with disease (Blauwendraat et al., 2020). PD is a complex neurodegenerative disorder displaying high heterogeneity in terms of clinical presentation, environmental and genetic factors contributing to this pathology (Ryden and Lewis, 2019). PD is defined as a chronic progressive movement disorder mainly characterized by resting tremor, rigidity and bradykinesia (Strafella et al., 2018; Ryden and Lewis, 2019). Patients can also experience non-motor symptoms (REM-sleep behavior disorder, cognitive, neuropsychiatric, sensory and autonomic disturbances) (Ryden and Lewis, 2019). Environmental factors affecting the onset and course of PD include smoking, pesticides, drug use, comorbidities (Strafella et al., 2018; Blauwendraat et al., 2020). The genetic contribution to disease consists of a wide spectrum of associated variants (Strafella et al., 2018; Nalls et al., 2019). Rare, highly penetrant variants are normally found in patients with monogenic familial PD (Strafella et al., 2018). Sporadic idiopathic forms of PD are frequently characterized by common variants, which individually play a small effect on the lifetime risk of disease (Blauwendraat et al., 2020). To date, up to 90 risk variants have been associated with sporadic PD (Nalls et al., 2019). Several biological pathways have been described in relation to PD pathophysiology, including cell-death, oxidative stress, immuno-inflammatory response, mitochondrial dysfunction, endocytosis, lysosomal-autophagy system, synaptic dysfunction, trafficking and toxic accumulation of α-Synuclein (αSYN) (Ryden and Lewis, 2019). In particular, genetic and expression studies described several genes involved in inflammation and immune system as risk factors for PD, supporting the existence of a link between such mechanisms and the etiology and progression of neurodegenerative processes (Hamza et al., 2010; Raj et al., 2014; Bossù et al., 2015; Billingsley et al., 2018; Garretti et al., 2019). It is generally accepted that the persistent activation of brain resident immune cells (i.e., glial cells) and loss of communication between glial cells and neurons results in the development of neuroinflammation, which is a crucial player in neurodegenerative diseases, including PD. However, extensive literature studies reviewed the contribution of PD causative genes (a-syn, Parkin and DJ-1) in innate immune responses, the immunoregulatory role of dopamine and the disruption of Blood-Brain-Barrier integrity in PD (Bossù et al., 2015; Troncoso-Escudero et al., 2018; Garretti et al., 2019; Matt and Gaskill, 2020). This last finding advocated for a role of peripheral immune system in neuroinflammation, through the recruitment of blood-derived immune cells and the release of inflammatory molecules (Louveau et al., 2015; Troncoso-Escudero et al., 2018). Altogether, these studies highlighted the need of further exploring the link between peripheral immune system and Central Nervous System (CNS) and the high therapeutic potential of neuroinflammation for treating neurodegenerative disorders, such as PD. In this context, the present study aimed at investigating the burden of variants falling within genes involved in the modulation of immune system, cellular degeneration and neuroinflammation, which may be provide insightful clues into the mechanisms underlying the susceptibility and progression of idiopathic PD. The study included a panel of 120 SNPs, which has been utilized in a previous work (Strafella et al., 2021, in press) focused on the study of such variants in another complex disorder (i.e., Age- Related Macular Degeneration, AMD) sharing several pathophysiological mechanisms with neurodegenerative disorders, including PD. Indeed, this study has been thought to extend the research of susceptibility and prognostic biomarkers of disease as well as for proposing additional targets for novel therapies or drug repurposing strategies for the treatment of PD.

## Materials and Methods

### Study Subjects

The study cohort involved 342 Italian unrelated patients affected by idiopathic PD recruited from the Laboratory of Neuropsychiatry of IRCCS Santa Lucia Foundation in Rome and IRCCS Neuromed in Pozzilli (Italy). The diagnosis of PD was performed according to the specific diagnostic criteria (Pellicano et al., 2017). The detailed patients’ characteristics are summarized in Table 1. In patients with positive family history for PD, the presence of known pathogenic mutations associated with monogenic forms of PD was excluded. The research was approved by the Ethical committee (CE/PROG.650 approved on 01/03/2018) of IRCCS Santa Lucia Foundation Hospital of Rome and was performed according to the Declaration of Helsinki. Written informed consent was obtained for all patients. The 503 control subjects were represented by European samples derived from 1,000 Genomes and GnomAD databases.

**TABLE 1:** Characteristics of PD patients.

**Study subjects**	**Age**	**Age of diagnosis**	**Age of onset**	**Disease duration**	**UPDRS III**	**Familiarity for PD (%)**
Female subjects	66.4 ± 8.43	60.4 ± 9.30	56.8 ± 9.25	9.62 ± 7.84	19.2 ± 14.2	42 (35.8)
Male subjects	66.6 ± 9.54	63.2 ± 9.36	58.9 ± 11.2	7.71 ± 7.62	19.4 ± 13.8	65 (28.9)
Total subjects	66.5 ± 9.17	62.3 ± 9.41	58.2 ± 10.6	8.36 ± 7.74	19.3 ± 13.9	107 (31)

*Detailed information about the PD patients recruited for the study are summarized according to the gender. Mean ± Standard Deviation (SD) are shown for all the features except for familiarity, which has been reported as percentage in the cohort. Motor symptoms were measured by means of Unified Parkinson’s Disease Rating Scale part III (UPDRS III).*

### Selection of Genetic Variants

The present study was conducted utilizing a panel of 120 variants already employed for a previous study (Strafella et al., 2021, in press) performed on another complex disorder, that share some of the pathophysiological mechanisms occurring in neurodegenerative diseases, including PD. A detailed description of the methods utilized for selecting the variants of interest is available within the referenced paper and its supplementary material (Strafella et al., 2021, in press).

The existence of Linkage Disequilibrium (LD) patterns among variants located on the same chromosomes was evaluated in European samples derived from 1,000 Genomes database. LD analysis was performed through the LDmatrix tool of LDlink software (Machiela and Chanock, 2015), obtaining a heatmap matrix representing the LD patterns among the variants for each chromosome. D′ and *R*^2^-values were obtained for each pairwise LD.

### DNA Extraction and Quantification

Genomic DNA was extracted from 200 to 400 μL of blood with MagPurix Blood DNA Extraction Kit and MagPurix Automatic Extraction System (Resnova, Italy) according to the manufacturer’s instructions. The concentration and quality of the extracted DNA have been assessed by DeNovix Spectrophotometer (Resnova, Italy). In particular, DNA samples were characterized by a concentration range of 50–150 ng/μL and A260/230 and A260/280 ratios included between 1.7 and 1.9.

### Genotyping Analysis

The genotyping analysis of the DNA samples was conducted by OpenArray Real-Time PCR on Quant Studio 12K Flex Real Time PCR System (Thermo Fisher Scientific, CA, United States). A customized panel of 120 assays specific for the selected variants enabled the simultaneous genotyping of 24 DNA samples per plate. For each sample, 30–150 ng of extracted DNA were re-suspended in 3μL of pure distilled water and manually loaded into 384 well-plates with 3μL of TaqMan OpenArray Genotyping Master Mix according to manufacturer’s instructions. Negative controls were obtained by mixing water and Master Mix (1:1). The mix were automatically transferred on the plates through the QuantStudio 12K Flex Accufill System and subsequently loaded into the QuantStudio 12K Flex Real Time PCR system (Thermo Fisher Scientific, CA, United States). Results were analyzed by the Taqman Genotyper Software (Thermo Fisher Scientific, CA, United States). Cluster normalization was performed with default parameters to normalize run-to-run variations in cluster positions caused by differences in reagent lots and experimental conditions. For each SNP, the call rate was evaluated considering a cut-off of 90%. Therefore, 15 SNP assays did not reach this threshold and thereby were excluded from further analyses. The removed variants were rs2925980, rs10074258, rs2672603, rs356219, rs17174870, rs228614, rs731236, rs6811520, rs12722489, rs1051643, rs45596840, rs2234975, rs1799983, rs786843, and rs4648356.

### Statistical Analysis

All the statistical analyses were performed with R software (R Core Team, 2020). Hardy-Weinberg Equilibrium (HWE) for the study cohort was tested by means of two-sided Fisher’s Exact test. The resulting data were considered in HWE with *p* > 0.05. As control group, genotype data of 503 European samples were extracted from Ensembl database. Two-sided Fisher’s Exact tests were calculated to compare allele and genotype frequencies of the selected variants between the two groups and perform statistical association analysis. The significance threshold was set at *p* < 0.05. In addition, the false discovery rate was evaluated by computing the *q*-value (q) and setting the significance cut-off at *q* < 0.05 (Storey et al., 2019). Alleles and genotypes Odds Ratios (ORs) and their 95% Confidence Interval (CI) were also estimated. The SNPs resulting significantly associated with PD were subjected to a logistic regression model considering the group as dependent variable. Bootstrap 632 + method (Efron and Tibshirani, 1997; Kuhn, 2020) in caret package was used for cross validation. The model performance was evaluated across resampling by averaging the Area Under the Receiver Operating Characteristics (AUROC), sensibility, specificity and accuracy metrics for each fold of the cross validation strategy (Kuhn, 2020).

Concerning the characteristics of PD patients, we performed a t-student and Fisher Exact test to assess the existence of statistical difference among male and female patients concerning the age, age of diagnosis, the age of onset, the duration of disease and motor symptoms (measured by Unified Parkinson’s Disease Rating Scale part III, UPDRS III) and familiarity. In particular, *t*-test was performed to compare mean values for age, age of diagnosis, age of onset, duration of disease and UPDRS III, whereas Fisher test was performed for testing frequency of familiarity in male and female patients, as it is a binary variable. Bonferroni correction was calculated to adjust *p* in both t-student and Fisher tests. Statistical thresholds of significance were fixed at *p* < 0.05.

The correlation analysis was performed by means of Pearson’s test and *T*-test in order to compare the variants co-occurrence in the same patient and clinical data. In particular, Pearson’s test was performed for numerical variables (age, age of diagnosis, age of onset, duration of disease and UPDRS III), whereas *T*-test was utilized for categorical variables. In both cases, the threshold of significance was set at *p* < 0.05.

LD patterns among the associated SNPs were evaluated in the PD cohort based on their location within the same chromosome. LD analysis was performed by Haploview 4.2 with default parameters, obtaining D′ and R^2^ scores for each pairwise LD (Barrett et al., 2005).

Genetic epistasis testing was performed among significant SNPs from Fisher’s exact test using *W*-test, which measures the association between binary phenotype and categorical genetic data (Sun et al., 2019). Obtained p were adjusted by means of Bonferroni correction with threshold set at *p* < 0.01 and number of categorical combination (*k* > 6) based on visual inspection of diagnostic plots (Sun et al., 2019).

### Bioinformatic Analyses

The significantly associated variants were tested for their potential effect on gene expression and function by means of bioinformatic tools. GTEx eQTL Calculator (GTEx Consortium, 2013) was used to evaluate the effect of each variant on the expression of the corresponding gene. In particular, variants distribution were analyzed on different brain areas (namely, Anterior Cingulate Cortex, ACC; Amygdala, AMY; Basal Ganglia, BG; Cortex, CX; Cerebellum, CE and Substantia Nigra, SN), considering *p* < 0.05 as significance threshold.

The variants located in the seed sequence of miRNAs were subjected to prediction analysis by ViennaRNAFold algorithm and PolymiRTS in order to evaluate the possible alteration of miRNAs biogenesis or binding affinity with target mRNAs, respectively. In particular, ViennaRNAfold algorithm allows predicting the secondary hairpin structures and computing the Minimum Free Energy (MFE, ΔG) that could affect the process of miRNAs biogenesis (Gruber et al., 2008). Wild-type and variant sequences of pre-miRNAs were retrieved from MiRBase (Griffiths-Jones et al., 2007). PolymiRTS predicted the impact of miRNA variants on miRNA-mRNA binding affinity through disruption or creation of binding sites (Bhattacharya et al., 2014). TargetScanHuman was interrogated to identify the genes targeted by the associated miRNAs (Agarwal et al., 2015). An *in silico* approach was implemented to analyze the differential expression of the associated miRNAs by extracting expression profiles of neuronal cells in control and sporadic PD patients from a public available small-RNA-seq study on GEO database (GSE110719) (Schulze et al., 2018). In particular, differential expression analysis was performed using DESeq2 (Love et al., 2014) and the base means across samples; the log^2^Fold Changes (Log^2^FC), standard errors (lfcSE), test statistics, p and adjusted p (adj-p) through Benjamini-Hochberg correction were calculated. The obtained results were plotted in form of a Vulcano plot.

The associated genes were used as input for Gene Set Enrichment Analysis (GSEA) on g:GOSt tool of g:Profiler online software (version e100_eg47_p14_7733820, Radvere et al., 2019) with default parameters (significance threshold method = g_SCS; user threshold = 0.05; sources = GO: Molecular Function, GO: Cellular Components, GO: Biological Processes, KEGG, Reactome, MIRNA, Human Protein Atlas, CORUM, Human Phenotype, WikiPathways). GSEA is a functional enrichment analysis on an input gene list. It maps genes to known functional information sources and detects statistically significantly enriched terms in the reference database by means of hypergeometric test. The results of this analysis were clustered on the basis of their semantic similarity using default parameters on Revigo (allowed similarity = 0.7; semantic similarity measure = SimRel) (Supek et al., 2011). The Ingenuity Pathway Analysis (IPA) and the generation of networks of interconnected genes and miRNA-target genes networks were performed on IPA software (Qiagen, CA, United States) and its related tools (Upstream Regulator Analysis, Disease and Functions and Path Designer). The software is backed by the Ingenuity Knowledge Base, which consists of highly structured, detail-rich biological and chemical findings. All the results generated by IPA software are referred as significant on the basis of the significance enrichment score fixed at *p* < 0.05 that is calculated by Fisher’s Exact Test. In the present study, we reported only significant results passing the significance threshold.

## Results

### Statistical Association Analysis

Results of the association analysis identified 26 SNPs significantly associated with a higher susceptibility to PD. In particular, all of the associated SNPs showed different frequency distributions between cases and controls, both at allelic (Table 2) and genotype level (Supplementary Table 1). Successively, the logistic regression analysis classified rs429358, rs1800795, rs2303759, rs3746444, rs190982, rs24168, and rs13401 as the strongest predictive variants for PD risk in the present study (Table 3). Consistent with this finding, the R^2^ (0.28), ROC (0.76), sensitivity (0.67), specificity (0.69) and accuracy (0.68) values confirmed the good reliability and performance of the logistic regression model.

**TABLE 2:** SNPs significantly associated with PD.

**SNP (gene)**	**Variant type**	**Allele count in cases (frequency)**	**Allele count in controls (frequency)**	***p*-value**	***q*-value**	**OR (95%CI)**
**rs429358, T/C (*APOE*)**	Exonic	T: 618 (0.93) C: 44 (0.07)	T: 850 (0.85) C:156 (0.15)	2.18*10^–8^	5.61*10*10^–7^	*T* = 2.57 (1.80–3.74)
**rs11218343, T/C (*SORL1*)**	Intron	T: 613 (0.99) C: 1 (0.001)	T: 963 (0.96) C: 43 (0.04)	2.90*10^–8^	5.61*10^–7^	*T* = 27.34 (4.6–1102)
**rs1800795, C/G (*IL6*)**	Intron	C: 203 (0.30) G: 477 (0.70)	C: 418 (0.42) G: 588 (0.58)	9.89*10^–7^	1.53*10^–5^	*G* = 1.66 (1.35–2.06)
**rs729022, C/T (*SYT11*)**	3′UTR	C: 158 (0.24) T: 506 (0.76)	C: 341 (0.34) T: 665 (0.66)	9.48*10^–6^	0.0001	*T* = 1.64 (1.35–2.06)
**rs2075650, A/G (*APOE*)**	Intron	A: 631 (0.93) G: 45 (0.07)	A: 874 (0.87) G: 132 (0.13)	1.60*10^–5^	0.0001	*A* = 2.11 (1.47–3.08)
**rs670139, G/T (*MS4A4E*)**	**3**′**UTR**	**G: 462 (0.71)** **T: 190 (0.29)**	**G: 611 (0.61)** **T: 395 (0.39)**	2.52*10^–^**^5^**	**0.0002**	***G* = 1.57** **(1.26–1.95)**
**rs2303759, T/G (*DKKL1*)**	**Exonic**	**T:454 (0.67)** **G: 224 (0.33)**	**T: 765 (0.76)** **G: 241 (0.24)**	4.92*10^–^**^5^**	**0.0003**	***G* = 1.58** **(1.26–1.96)**
**rs874628, A/G (*MPV17L2*)**	**Exonic**	**A:422 (0.62)** **G:254 (0.38)**	**A: 723 (0.72)** **G: 283 (0.42)**	5.03*10^–^**^5^**	**0.0003**	***G* = 1.54** **(1.25–1.92)**
**rs3746444, A/G (*MIR499A*)**	**3**′**UTR**	**A: 487 (0.73)** **G: 183 (0.27)**	**A: 811 (0.81)** **G: 195 (0.19)**	**0.0001**	**0.001**	***G* = 1.56** **(1.23–2.0)**
**rs1077667 C/T (*TNFSF14*)**	Intron	C: 561 (0.84) T: 105 (0.16)	C: 776 (0.77) T: 230 (0.23)	0.0003	0.002	*C* = 1.58 (1.21–2.06)
**rs2283792, T/G (*MAPK1*)**	Intron	T: 262 (0.39) G: 406 (0.61)	T: 483 (0.48) G: 523 (0.52)	0.0004	0.002	*G* = 1.43 (1.16–1.75)
**rs12368653, G/A (*AGAP2*)**	**Intergenic**	**G: 286 (0.43)** **A: 386 (0.57)**	**G:511 (0.51)** **A: 495 (0.49)**	**0.0009**	**0.005**	***A* = 1.40** **(1.14–1.72)**
**rs190982, G/A (*MEF2C*)**	Intron	G:302 (0.45) A: 368 (0.55)	G: 373 (0.37) A: 633 (0.63)	0.001	0.005	*A* = 1.40 (1.13–1.72)
**rs2546890, A/G (*IL12B*)**	Non-coding transcript exon variant	A: 281 (0.41) G: 401 (0.59)	A: 494 (0.49) G. 512 (0.51)	0.001	0.006	*G* = 1.37 (1.12–1.68)
**rs24168, A/G (*MIR29A*)**	3′UTR	A: 316 (0.47) G: 346 (0.52)	A: 403 (0.40) G: 603 (0.60)	0.002	0.008	*A* = 1.36 (1.12–1.69)
**rs10466829, G/A (*CLECL1*)**	**Intron**	**G: 297 (0.43)** **A: 383 (0.56)**	**G: 515 (0.51)** **A: 491 (0.49)**	**0.002**	**0.01**	***A* = 1.35** **(1.11–1.66)**
**rs6964, G/A (*GAK*)**	3′UTR	G: 395 (0.61) A: 247 (0.38)	G: 691 (0.69) A: 315 (0.31)	0.003	0.01	*A* = 1.38 (1.11–1.72)
**rs2724377, A/G (*MIR29C*)**	3′UTR	A: 383 (0.58) G: 271 (0.41)	A: 519 (0.52) G: 487 (0.48)	0.005	0.01	*A* = 1.32 (1.08–1.62)
**rs7200786, A/G (*CLEC16A*)**	**Intron**	**A: 285 (0.42)** **G: 393 (0.58)**	**A: 491 (0.49)** **G: 515 (0.51)**	**0.007**	**0.02**	***G* = 1.31** **(1.07–1.60)**
**rs13401, G/A (*ATF6*)**	3′UTR	G: 188 (0.28) A: 484 (0.72)	G: 225 (0.22) A: 781 (0.77)	0.009	0.03	*G* = 1.34 (1.07–1.72)
**rs11614913, C/T (*MIR196A2*)**	**3**′**UTR**	**C: 431 (0.65)** **T: 229 (0.35)**	**C: 593 (0.59)** **T: 413 (0.41)**	**0.01**	**0.03**	***C* = 1.31** **(1.06–1.61)**
**rs755622, G/C (*MIF*)**	5′UTR	G: 568 (0.86) C: 92 (0.14)	G: 819 (0.81) C: 187 (0.19)	0.01	0.03	*G* = 1.40 (1.07–1.87)
**rs6897932, C/T (*IL7R*)**	**Exonic**	**C: 533 (0.78)** **T: 149 (0.22)**	**C: 733 (0.73)** **T: 273 (0.27)**	**0.01**	**0.03**	***C* = 1.33** **(1.05–1.68)**
**rs35349669, C/T (*INPP5D*)**	**Intron**	**C: 407 (0.60)** **T: 271 (0.40)**	**C: 543 (0.54)** **T: 463 (0.46)**	**0.01**	**0.03**	***C* = 1.28** **(1.04–1.56)**
**rs3745453, A/G** **(*ZSWIM4*)**	**3**′**UTR**	**A: 481 (0.73)** **G: 175 (0.27)**	**A: 683 (0.68)** **G: 323 (0.32)**	**0.01**	**0.04**	***A* = 1.29** **(1.03–1.62)**
**rs9331896, C/T (*CLU*)**	Intron	C: 228 (0.34) T: 444 (0.66)	C: 398 (0.40) T: 608 (0.60)	0.02	0.04	*T* = 1.27 (1.03–1.57)

*Novel SNPs (Genes) are highlighted in bold. OR, Odd Ratio; CI, Confidence Interval.*

**TABLE 3:** Logistic regression analysis on the SNPs associated with PD.

**SNP (gene)**	***p*-value**	**OR (95%CI)**
rs429358, T/C (*APOE*)	0.001	*T* = 5.0 (2.0–14.0)
rs1800795, C/G (*IL6*)	0.007	*G* = 2.0 (1.20–3.33)
rs2303759, T/G (*DKKL1*)	0.014	*G* = 1.86 (1.13–3.08)
rs3746444, A/G (*MIR499A*)	0.004	*G* = 2.09 (1.26–3.48)
rs190982, G/A (*MEF2C*)	0.003	*G* = 2.18 (1.28–3.68)
rs24168, A/G (*MIR29A*)	0.018	*A* = 1.89 (1.11–3.20)
rs13401, G/A (*ATF6*)	0.019	*G* = 1.80 (1.10–2.96)

*The SNPs with a p < 0.05 have been reported. OR, Odd Ratio; CI, Confidence Interval.*

Regarding the evaluation of difference concerning the characteristics of PD patients, only the duration of disease revealed a statistical difference between male and female patients (*p* = 0.001, corrected *p* = 0.006; Supplementary Table 2), with female patients showing a longer duration of disease with respect to males. However, nor the duration nor the other clinical data referred to patients were correlated with a statistical different genetic variants load (Supplementary Table 3).

### Evaluation of LD Patterns of Genetic Epistasis Among the SNPs Associated With PD Risk

We evaluated the LD patterns for the SNPs located on the same chromosome in order to search for different LD patterns between cases and control samples, which may affect the susceptibility to PD. As expected, the LD between rs429358 and rs2075650 (both located into *APOE*) showed comparable LD scores in PD samples (D′:0.74, R2:0.5) and control samples (D′:0.76, R2:0.48). None of the other variants located on the same chromosome were in LD meaning that they represent independent susceptibility markers for PD.

**FIGURE 1:**
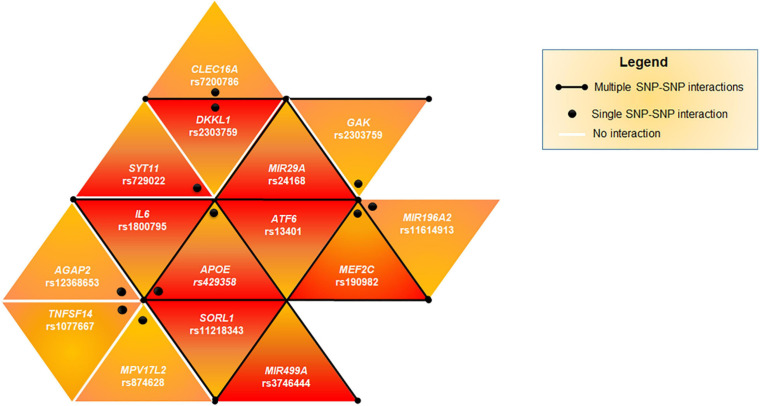
Genetic epistasis. The triangular network shows the epistatic interactions among the associated SNPs. Black lines highlights multiple SNPs interacting together (*APOE*, *MEF2C*, *ATF6*, *SORL1*, *IL6*, *DKKL1*, *GAK*, *MIR29A*, and *MIR499A*). Black dots indicate that the epistatic interaction affect one single SNP-SNP couple (*APOE*-*SYT11*, *APOE*-*AGAP2*, *APOE*-*TNFSF14*, *APOE*-*MPV17L2*, *DKKL1*-*CLEC16A*, and *MEF2C*-*MIR196A2*). The darker triangles show the most significant (*p* < 0.001) SNP-SNP interactions whereas the lighter ones displayed a lower significant (*p* < 0.01) interaction.

Successively, we evaluated the potential existence of genetic epistasis among the SNPs of interest. The analysis reported significant results for 16 SNPs out of 26 variants associated with PD (Supplementary Table 4). The interaction between the SNPs of *APOE* was excluded because they are in LD. The other interactions observed for 15 SNPs have been represented as a triangular network showing the different SNP-SNP interactions in Figure 1. The darker triangles show the most significant (*p* < 0.001) interactions whereas the lighter ones displayed the less significant (*p* < 0.01). In particular, rs429358 (*APOE*) and rs190982 (*MEF2C*) presented the highest number of interactions, suggesting their crucial role as key epistatic modulators. This finding suggests that, these genes should be regarded as a single complex network of epistatic interactions, providing an additional mechanism by which variants and genes may affect PD pathophysiology and susceptibility.

### eQTLs Analysis

**FIGURE 2:**
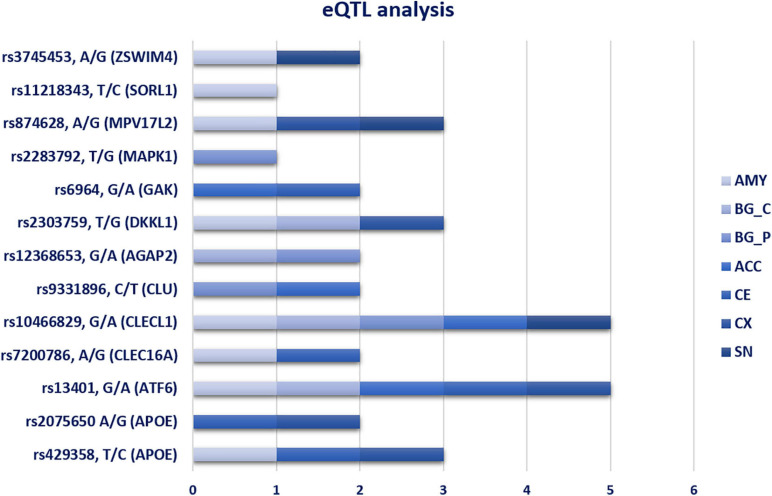
eQTL analysis in seven different brain tissues. The bar chart show the distribution of significant eQTL variants in the different brain tissues. AMY, Amygdala; BG_C, Basal Ganglia_Caudate; BG_P, Basal Ganglia_Putamen; ACC, Anterior Cingulate Cortex; CE, Cerebellum; CX, cortex; SN, Substantia Nigra.

The analysis of the 26 SNPs associated with PD as potential eQTLs provided significant results for 12 variants, whose genotypes affected the expression of their related-genes. These variants were found to be differentially associated in different brain tissues, namely ACC, AMY, BG, CE, CX, and SN (Figure 2). Among the 12 eQTL variants, rs13401 (*ATF6*) and rs10466829 (*CLECL1*) appeared to be significantly associated in five brain tissues (Figure 2), suggesting that these variants are likely to affect gene functions that are important for the brain as a whole. In contrast, rs2075650 (*APOE*), rs7200786 (*CLEC16A*), rs9331896 (*CLU*), rs6964 (*GAK*), rs2283792 (*MAPK1*), rs11218343 (*SORL1*), and rs3745453 (*ZSWIM4*) were distributed in specific regions of the brain (Figure 2), indicating that these variants may exert their regulatory role in genes expressed in specific brain areas.

### Functional Analysis of Variants Located in miRNA-Genes

Four variants located into four miRNA-genes were associated with PD in the present study, namely rs24168, rs2724377, rs11614913, and rs3746444. The rs11614913 (*MIR196A2*) and rs3746444 (*MIR499A*) were located in the seed sequence of miR-196a2 and miR-499a. Both SNPs were therefore subjected to prediction analysis by ViennaRNAFold algorithm, and PolymiRTS and TargetScanHuman tools.

Concerning the rs11614913 (*MIR196A2*, C/T), the secondary structure generated by the sequence carrying the risk allele (C) was characterized by a lower MFE (-50.30 Kcal/mol) compared to the structure containing the T allele (MFE = -44.70 Kcal/mol). The different MFE observed in the hairpin structure carrying the risk allele may enhance the stability of the pre-miR-196a2 and the subsequent processing and expression of the mature miR-196a. The prediction analysis of the binding affinity of miR-196a2 by PolymiRTS was not possible because the miRNA and the variant were not annotated on the database. However, the prediction of the PD-associated genes targeted by miR-196a2 by TargetScanHuman revealed that miR-196a2-3p may target *SYT11*, *MAPK1*, *MEF2C*, *IL12B*, *ATF6*, *IL7R*, and *CLU*. The risk allele (C) of rs11614913 may therefore enhance the expression of miR-196a which, in turn, could affect the expression of its target genes and their related biological pathways involved in PD. Notably, the analysis of the expression profile of miR-196a in neuronal cells of PD patients derived by public available small-RNA-seq data (GSE110719) (Schulze et al., 2018), showed an increased expression (log2 Fold Change = 1.61) of miR-196a-3p in PD cells with respect to control samples (Supplementary Figure 1).

Concerning the rs3746444 (*MIR499A*, A/G), the comparison of the secondary structures generated by the sequence variations and the MFE did not report significant differences. However, interrogation of PolymiRTS showed that the risk allele (G) is predicted to disrupt the binding sites for *MEF2C* and *INPP5D* genes, losing the ability of modulating their expression. The TargetScanHuman prediction analysis reported that miR-499a-3p may target *TNFSF14*, *MAPK1*, *MEF2C*, *ATF6*, *MAOA*, *IL7R*, and *INPP5D*. These findings suggested that miR-499a could be implicated in PD pathophysiology by modulating the expression of its target genes and the associated pathways. Interestingly, we found an increased expression of miR-499a-3p (log^2^ Fold Change = 2.15) in neuronal samples of PD patients compared to controls (Supplementary Figure 1). The samples for this analysis have been retrieved by public available small-RNA-seq data (GSE110719) (Schulze et al., 2018).

The rs24168 (*MIR29A*) and rs2724377 (*MIR29C*) are located outside the stem-loop region (pre-miRNA region) and therefore could not be subjected to the prediction analysis for evaluating their impact miRNA biogenesis and binding affinity. Interrogation of TargetScanHuman reported that miR29a-5p may target *TNFSF14*, *MAPK1*, *ATF6*, *IL7R*, and *INPP5D*, whereas any of the PD-associated genes in this study resulted to be a target of miR29c.

### Gene Ontology (GO) Enrichment Analysis and Ingenuity Pathway Analysis (IPA)

**FIGURE 3:**
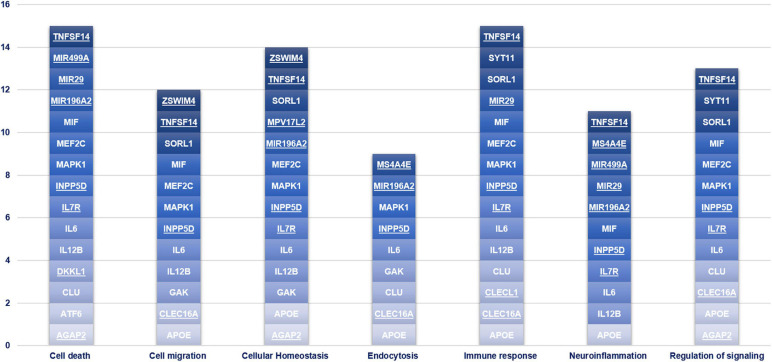
Gene Set enrichment analysis. This chart illustrates the main biological pathways affected by the genes associated with PD in the present study. The novel susceptibility genes identified in the present study are underlined.

**FIGURE 4:**
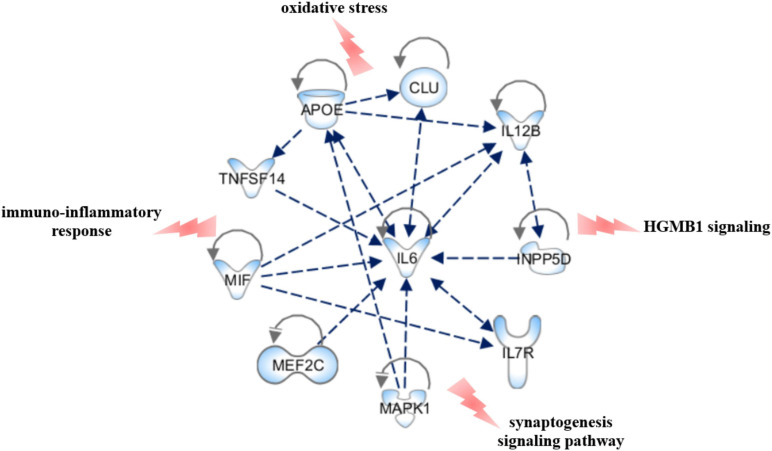
Upstream regulator analysis. The figure shows the PD-associated genes that have been predicted to act as upstream regulators by IPA software and illustrates how they may interact together and affect their mutual expression. The canonical pathways that may be affected by the network have also been reported. The figure has been created by Path Designer tool available on IPA software (Qiagen).

We performed Gene Set Enrichment Analysis (GSEA) by Revigo and g:Profiler webservers and the Ingenuity Pathway Analysis by IPA software application (Qiagen) to find a possible link between the associated genes and the biological and pathophysiological mechanisms underlying PD. The GSEA and IPA allowed identifying endocytosis, apoptosis, migration of cells, signal transduction, immuno-inflammatory response and cellular homeostasis as the main biological pathways affected by the associated genes (Figure 3). Successively, we assessed which of the genes associated with PD in this study may affect their mutual expression by a tool available on IPA. This analysis revealed that *APOE*, *CLU*, *IL6*, *IL7R*, *IL12B*, *INPP5D*, *MAPK1*, *MEF2C*, *MIF*, and *TNFSF14* may act as upstream regulators and mutually alter their expression (Figure 4). This result indicates that these genes should be regarded as a network of interconnected genes working altogether in the modulation of biological pathways implicated in the onset and progression of PD. It was not possible to predict in which direction (inhibition or activation) the observed genes may regulate the expression activity, as expression data are not available nor at the laboratory nor as public available RNA-seq data for our genes of interest. However, this analysis revealed how the associated genes may interact together and contribute to PD pathophysiology and highlighted IL6 as the master gene regulator of this network, as it either can affect the expression of the other associated genes or be targeted by them.

## Discussion

The present study aimed at investigating the burden of genetic variants located within genes involved in immune system response, cellular degeneration and neuroinflammation, which may improve the understanding of the mechanisms affecting the susceptibility and progression to idiopathic PD. Therefore, a panel of variants utilized in a previous work focusing on the study of similar processes in another complex disorder was employed, with the purpose of extending the knowledge of variants and genes which are likely to affect the susceptibility and pathophysiology of PD at systemic and brain/neuronal level. As a result, 26 SNPs out of 120 investigated variants were significantly associated with idiopathic PD (Table 2). The logistic regression analysis identified seven SNPs as the strongest predictive markers for PD risk (Table 3) suggesting that these variants may be exploited for developing genetic risk models able to stratify at-risk individuals who may benefit of early diagnosis and therapeutic treatments. Moreover, statistical analysis revealed a significant difference among male and female patients in relation to the duration of disease (Supplementary Table 2), with the females having a longer duration of disease with respect to males (Table 1). This result is consistent with literature, referring that woman have not only longer disease duration but also more severe progression and lower response to therapeutic treatment (Picillo et al., 2017; Jurado-Coronel et al., 2018). However nor the disease duration nor the other tested variable were significantly correlated with a different variant load (Supplementary Table 3), indicating that the associated SNPs were independent susceptibility factors for PD.

Interestingly, many associated SNPs displayed the highest frequent allele associated with PD risk. This result is consistent with the fact that idiopathic PD is a multifactorial disorder whose susceptibility also depends from environmental and lifestyle conditions that frequently change over time and can affect the natural selection of genetic variants and the susceptibility to different diseases (Kido et al., 2018). Genetic variants, which may have been selected in the past because of their neutral or low impact on protein, may have become risk alleles because of the modern environment and lifestyle changes (Cascella et al., 2015; Gorlov et al., 2015; Kido et al., 2018). Similar to PD, frequent risk alleles have been often observed in other complex disorders (Kido et al., 2018; Buniello et al., 2019).

The analysis of genetic epistatic effects among the associated SNPs provided significant interactions for 15 SNPs (Figure 1), suggesting that they may contribute to PD susceptibility by synergistic interactions because of genetic epistasis. This network of epistatic interactions may be useful for setting-up a genetic risk model for PD, which accounts not only for genetic variants with a cumulative effect-size but also for variants contributing to disease susceptibility by synergistic effects.

In this study, 11 SNPs associated with PD mapped to genes (*MS4A4E*, *DKKL1*, *MPV17L2*, *MIR499A*, *AGAP2*, *CLECL1*, *CLEC16A*, *MIR196A2*, *IL7R*, *INPP5D*, and *ZSWIM4*) which have never been investigated in PD to our knowledge. These novel susceptibility genes encode proteins essentially involved in pathways related to PD, including neuroinflammation (MS4A4E and INPP5D) (Malik et al., 2015; O’Day, 2019), peripheral immune response (CLECL1, CLEC16A, IL7R, MS4A4E, and INPP5D) (Cukier et al., 2017; O’Day, 2019), apoptosis (AGAP2, DKKL1) (Wu et al., 2013; Workman et al., 2018), autophagy (CLEC16A) (Tam et al., 2017), endo-lysosomal system (AGAP2 and MS4A4E) (Wu et al., 2013), mitochondrial function/morphology (MPV17L2) (Dalla Rosa et al., 2014) and axon guidance (ZSWIM4) (Fink et al., 2017). In particular, this study confirmed the involvement of peripheral immune response in PD susceptibility and progression. Interestingly, most of the proteins (CLECL1, CLEC16A, MS4A4E, and INPP5D) affecting the peripheral immune response are heavily expressed in dendritic cells, which play a major role in the immuno-inflammatory processes leading to neuroinflammation and neurodegeneration in PD (Bossù et al., 2015; Troncoso-Escudero et al., 2018).

Overall, the genetic variants within the novel associated genes may contribute to PD susceptibility by altering multiple signaling pathways, which could be further explored by functional studies.

Most of the associated SNPs were located within intronic and 3′UTR regions. This finding suggested that their genetic contribution to PD susceptibility may be explicated by the alteration of regulatory elements that normally modulate gene expression. In fact, eQTLs analysis reported 12 significant variants distributed in multiple brain regions (Figure 2). These variants may exert a different regulatory role according to the brain-affected area and, consequently, may be associated with motor and non-motor PD phenomenology. Studying the eQTL variants allocated in BG (Caudate and Putamen), CX and CE would be extremely important to understand their role in the alteration of biological pathways crucially involved in PD phenomenology, especially concerning motor and cognitive symptoms (tremor, freezing, action sequencing impairment) (Caligiore et al., 2016). In addition, resting tremor is primarily due to the death of dopaminergic neurons in the SN compacta (SNc) (Caligiore et al., 2016). Concerning the eQTLs variants in AMY and ACC, they may help to link the alteration of gene expression in these regions with PD non-motor symptoms, especially cognitive impairments and Impulse Control Disorders (ICDs) (Caligiore et al., 2016).

**FIGURE 5:**
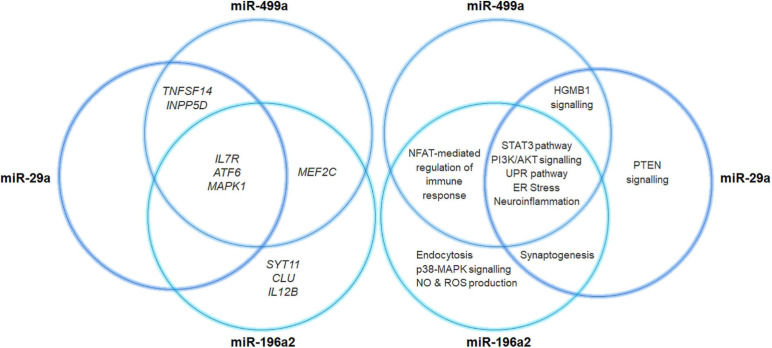
Venn diagram illustrating miRNAs-target gene networks. The diagram illustrates the interaction of miR29a, miR196a, and miR499a with their related target genes. The canonical pathways that may be affected by the miRNA-target gene interactions have also been reported.

Regarding the relationship between miRNA variants and PD, this study allowed identifying two novel miRNAs (miR-499a and miR-196a2) associated with PD and confirming the known association of miR-29a and miR-29c. The SNPs located within *MIR29A* and *MIR29C* were never been investigated in PD, although downregulated expression of both miRNAs have been associated with the disease in literature (Bai et al., 2017; Wang et al., 2020). Concerning the variants located in *MIR196A2* and *MIR499A*, they were found to affect the biogenesis and binding affinity of their corresponding miRNAs, respectively. Statistical and bioinformatic approaches showed that miRNA variants and their related-miRNAs could closely cooperate with multiple genes through epistatic interactions or by participating into different biological pathways. Moreover, the analysis of a public available RNA-seq data revealed an increased expression of miR-196a and miR-499a in neuronal samples derived from PD patients compared to controls (Supplementary Figure 1). As illustrated in Figure 5, the associated miRNAs target several associated genes, which are involved in a number of signaling pathways (namely HGMB1, p38MAPK, PTEN, and PI3K/AKT, ERS and UPR) specifically involved in the modulation of immune response, neuroinflammation and neurodegeneration reported in PD (Jha et al., 2015; Angelopoulou et al., 2018; Joe et al., 2018; Liu et al., 2019; Martinez et al., 2019). Altogether, the study of miRNA-gene target interactions advocate for a role of miR-499a, miR-196a2, and miR-29a in the modulation of multiple neuroinflammatory and neurodegenerative mechanisms underlying PD etiopathogenesis and progression, supporting thereby their possible therapeutic potential.

The present study highlighted *IL-6* as the master gene regulator of a network of interconnected genes (Figure 4), which may cooperate altogether to modulate several biological pathways implicated in PD neuroinflammatory and neurodegenerative mechanisms. It is a matter of fact that IL6 takes part in multiple intracellular signaling pathways mainly involved in the immunoinflammatory response in several tissues, including the brain (Garbers et al., 2018; Murakami et al., 2019). Several studies highlighted the association of genetic variants in *IL6* with the onset and progression of several complex disorders, mediated by neuroinflammation and immune system dysfunction (Woo and Humphries, 2013; Mun et al., 2016; Redenšek et al., 2019; Strafella et al., 2020, 2021). These data, together with the evidence of association between high IL6 levels and neuroinflammation support IL6 as a promising drug target for PD and other neurodegenerative diseases mediated by neuroinflammation (Garbers et al., 2018). Indeed, several drugs targeting IL6 and its receptor (IL6R) currently used for the treatment of autoimmune or inflammatory disorders (Choy et al., 2020) may be further investigated for potential drug repurposing strategies in the management of PD and other neurodegenerative pathologies. In addition, it is worth to further investigate IL6 as a possible prognostic biomarker for PD, which may be useful for the implementation of more suitable treatment strategies aimed to improve patients’ prognosis and quality of life considering the disease trajectory.

Altogether, the present study highlighted several genetic association and miRNA-target gene interactions, which could be involved in PD physiopathology. The variants, the genes and miRNAs discussed in this study emphasize the possible role of peripheral, cellular and molecular players in determining the susceptibility to develop PD, with the immune response and cell death pathways representing the top contributors (Figure 3) in this study. Indeed, this study highlighted once again the high therapeutic potential of neuroinflammation for PD treatment, given the direct or indirect involvement of the associated genes illustrated in this study. However, future functional studies tailored to investigate the disease by multilayer approaches are expected, in order to clarify the effective relationship between peripheral and neuronal players in the etiopathogenesis/progression of disease, and to translate research results into the design of more effective lines of treatments for PD. Indeed, a deeper exploration of the above-discussed interactions could inspire future research for novel prognostic biomarkers as well as novel therapeutic or drug repurposing strategies for the management and treatment of the disease.

## Data Availability Statement

All data generated and supporting this study are included within the manuscript and its Supplementary Material.

## Ethics Statement

The studies involving human participants were reviewed and approved by the Ethics Committee of IRCCS Santa Lucia Foundation Hospital of Rome. The patients/participants provided their written informed consent to participate in this study.

## Author Contributions

CS, VC, RC, and EG conceived the study and designed the experiments. VC performed the experiments. AT performed statistical analyses wrote code, ran the model, and analyzed output data. CS, VC, and AT performed bioinformatic analyses. CS, VC, AT, GM, and RC interpreted the obtained data. FA, CP, FP, LM, SG, DC, PB, GS, and CC were engaged of the collection and acquisition and management of clinical data. CS, VC, AT, and RC wrote the manuscript. CS, GM, DC, PB, GS, CC, RC, and EG performed the critical revision and editing of the manuscript. RC and EG supervised the work. All authors read and approved the final manuscript.

## Conflict of Interest

The authors declare that the research was conducted in the absence of any commercial or financial relationships that could be construed as a potential conflict of interest.

## Supplementary Material

Supplementary Figure 1 Vulcan Plot reporting the results of miR-196a2 and miR499a expression analysis using public available RNA-seq data (GSE110719).

Supplementary Table 1 Association analysis of the risk genotypes associated with PD.

Supplementary Table 2 Assessment of statistical difference between male and female patients by t-test and fisher-test. Significance threshold were fixed at *p* < 0.05.

Supplementary Table 3 This table reports the results for the correlation tests between variants co-occurrence in the same patient and clinical data. Conf. Low, Lower Confidence Interval; Conf. High, Higher Confidence Interval.

Supplementary Table 4 Results concerning the significant epistatic interactions for the SNPs of interest.
