# Supplementary figures and images for: Immune System and Neuroinflammation in Idiopathic Parkinson’s Disease: Association Analysis of Genetic Variants and miRNAs Interactions
Source: Front Genet. 2021 Jun 3;12:651971. doi: 10.3389/fgene.2021.651971 (PMC8209518; doi:10.3389/fgene.2021.651971)

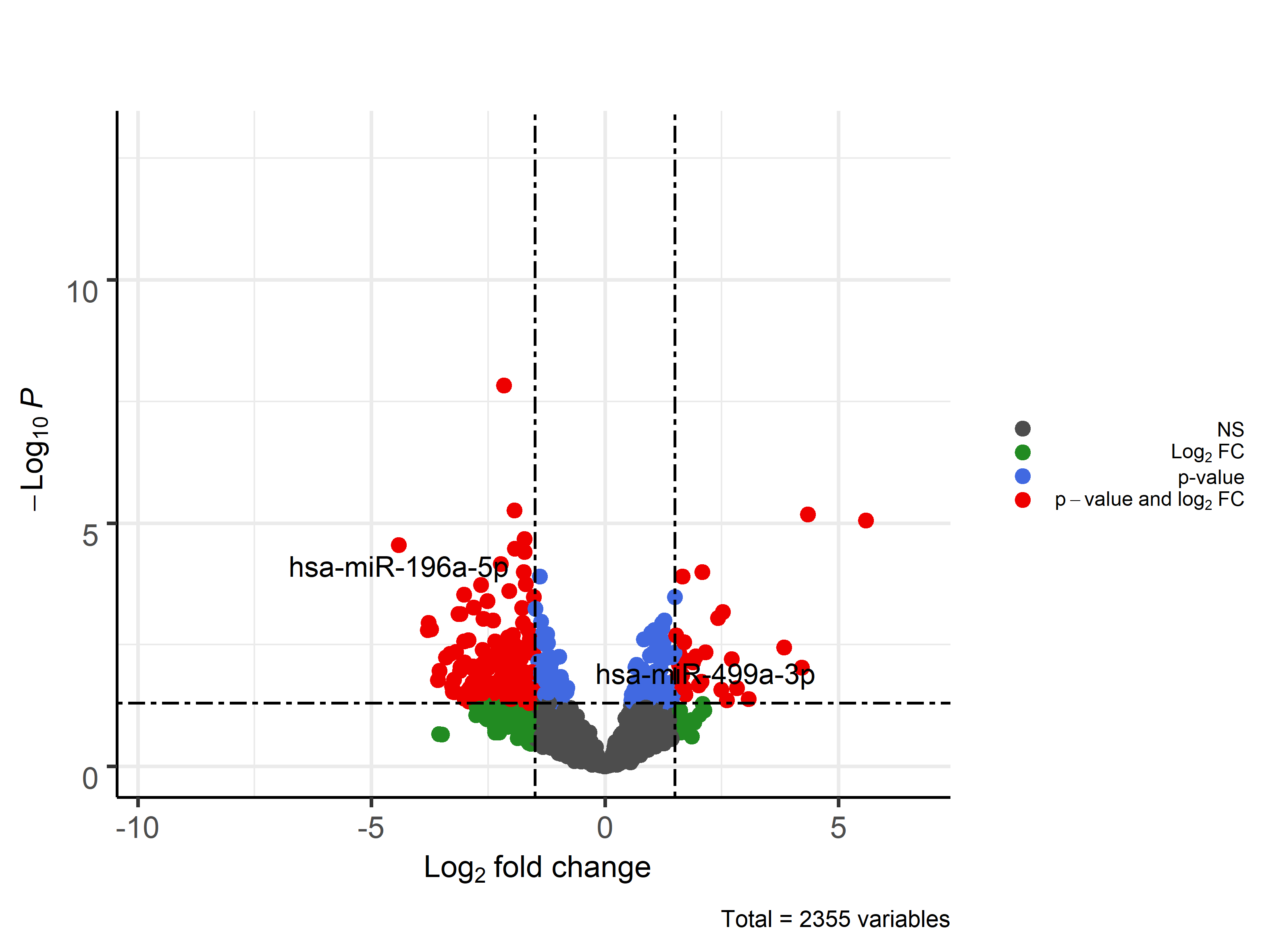

Supplement: Supplementary Figure 1 — Vulcan Plot reporting the results of miR-196a2 and miR499a expression analysis using public available RNA-seq data (GSE110719). [file Image_1.PNG]
