# Supplementary material for: Immune System and Neuroinflammation in Idiopathic Parkinson’s Disease: Association Analysis of Genetic Variants and miRNAs Interactions
Source: Front Genet. 2021 Jun 3;12:651971. doi: 10.3389/fgene.2021.651971 (PMC8209518; doi:10.3389/fgene.2021.651971)
Supplement: Supplementary Table 1 — Association analysis of the risk genotypes associated with PD. [file Table_1.DOCX]

**Supplementary Table 1.** Genotype association analysis of the SNPs associated with PD.

OR: Odd Ratio, CI: Confidence Interval.

| *SNP* (*Gene*) | Genotype counts in cases (Frequency) | Genotype counts in controls (Frequency) | *p-value* | *q-value* | OR (95%CI) |
| --- | --- | --- | --- | --- | --- |
| rs429358, T/C (*APOE*) | TT: 289 (0.87)  TC: 40 (0.13)  CC: 2 (0.006) | TT: 358 (0.72)  TC: 134 (0.26)  CC: 11 (0.02) | 7.58*10^-8^ | 6.07*10^-7^ | TC= 2.70 (1.81-4.16)  TT= 4.54 (1.00-50.0) |
| rs11218343, T/C (*SORL1*) | TT: 306 (0.99)  TC: 1 (0.01)  CC: 0 (0.00) | TT: 462 (0.92)  TC: 39 (0.08)  CC: 2 (0.004) | 9.42*10^-8^ | 6.07*10^-7^ | TC= 33.33(4.34-1111.0)  TT= ∞ |
| rs1800795, C/G (*IL6*) | CC: 38 (0.11)  CG: 127 (0.37)  GG: 175 (0.52) | CC: 96 (0.19)  CG: 226 (0.45)  GG: 181 (0.36) | 1.44*10^-5^ | 6.21*10^-5^ | CG= 1.72 (1.26-2.38)  GG= 2.43 (1.56-4.0) |
| rs729022, C/T (*SYT11*) | CC: 27 (0.08)  CT: 104 (0.31)  TT: 201 (0.61) | CC: 65 (0.13)  CT: 211 (0.42)  TT: 227 (0.45) | 5.97*10^-5^ | 0.00021 | CT= 1.81 (1.31-2.50)  TT= 2.17 (1.29-3.70) |
| rs2075650, A/G (*APOE*) | AA: 296 (0.88)  AG: 39 (0.11)  GG: 3 (0.01) | AA: 382 (0.76)  AG: 110 (0.22)  GG: 11 (0.02) | 9.42*10^-5^ | 0.0003 | AG= 2.22 (1.47-3.44)  AA= 2.85 (1.00-16.66) |
| rs670139, G/T (*MS4A4E*) | GG: 171 (0.52)  GT: 120 (0.38)  TT: 35 (0.10) | GG: 193 (0.38)  GT: 225 (0.45)  TT: 85 (0.17) | 0.0001 | 0.0004 | GT= 1.66 (1.21-2.32)  GG= 2.17 (1.36-3.57) |
| rs2303759, T/G (*DKKL1*) | TT: 150 (0.44)  TG: 154 (0.46)  GG: 35 (0.10) | TT: 294 (0.58)  TG: 177 (0.35)  GG: 32 (0.06) | 0.0001 | 0.0004 | TG= 1.70 (1.25-2.30)  GG= 2.14 (1.23-3.72) |
| rs874628, A/G (*MPV17L2*) | AA: 137 (0.40)  AG: 148 (0.44)  GG: 53 (0.16) | AA: 258 (0.51)  AG: 207 (0.41)  GG: 38 (0.08) | 0.0001 | 0.0004 | AG= 1.34 (1.00-1.82)  GG= 2.62 (1.60-4.30) |
| rs3746444, A/G (*MIR499A*) | AA: 176 (0.53)  AG: 135 (0.40)  GG: 24 (0.07) | AA: 330 (0.65)  AG: 151 (0.30)  GG: 22 (0.04) | 0.0006 | 0.001 | AG= 1.67 (1.23-2.27)  GG= 2.04 (1.06-3.94) |
| rs1077667 C/T (*TNFSF14*) | CC: 235 (0.71)  CT: 91 (0.27)  TT: 7 (0.02) | CC: 303 (0.60)  CT: 170 (0.34)  TT: 30 (0.06) | 0.001 | 0.002 | CT= 1.44 (1.06-1.81)  CC= 3.33 (1.40-10.0) |
| rs2283792, T/G (*MAPK1*) | TT: 51 (0.15)  TG: 160 (0.48)  GG: 123 (0.37) | TT: 127 (0.25)  TG: 229 (0.46)  GG: 147 (0.29) | 0.001 | 0.002 | TG= 1.20 (1.00-1.66)  GG= 2.08 (1.36-3.22) |
| rs12368653, G/A (*AGAP2*) | GG: 63 (0.19)  GA: 160 (0.48)  AA: 113 (0.33) | GG: 124 (0.25)  GA: 263 (0.52)  AA: 116 (0.23) | 0.001 | 0.02 | GA= 1.19 (1.00-1.75)  AA= 1.91 (1.26-2.91) |
| rs190982, G/A (*MEF2C*) | GG: 66 (0.20)  GA: 170 (0.51)  AA: 99 (0.29) | GG: 78 (0.15)  GA: 217 (0.43)  AA: 208 (0.41) | 0.002 | 0.003 | GA= 1.64 (1.19-2.27)  GG= 1.77 (1.15-2.72) |
| rs2546890, A/G (*IL12B*) | AA: 59 (0.17)  AG: 163 (0.48)  GG: 119 (0.35) | AA: 125 (0.25)  AG: 244 (0.48)  GG: 134 (0.27) | 0.006 | 0.007 | AG= 1.33 (1.00-1.85)  GG= 1.88 (1.25-2.85) |
| rs24168, A/G (*MIR29A*) | AA: 83 (0.25)  AG: 150 (0.45)  GG: 98 (0.30) | AA: 88 (0.18)  AG: 227 (0.45)  GG: 188 (0.37) | 0.01 | 0.01 | AG= 1.26 (1.00-1.76)  AA= 1.80 (1.20-2.71) |
| rs10466829, G/A (*CLECL1*) | GG: 71 (0.21)  GA: 155 (0.46)  AA: 114 (0.33) | GG: 132 (0.26)  GA: 251 (0.50)  AA: 120 (0.24) | 0.006 | 0.007 | GA= 1.14 (1.00-1.65)  AA= 1.76 (1.17-2.64) |
| rs6964, G/A (*GAK*) | GG: 133 (0.41)  GA:129 (0.41)  AA:59 (0.18) | GG: 241 (0.48)  GA: 209 (0.42)  AA: 53 (0.10) | 0.005 | 0.006 | GA= 1.11 (1.00-1.53)  AA= 2.01 (1.28-3.16) |
| rs2724377, A/G (*MIR29C*) | AA: 111 (0.34)  AG: 161 (0.49)  GG: 55 (0.17) | AA: 130 (0.26)  AG: 259 (0.51)  GG: 114 (0.23) | 0.01 | 0.01 | AG= 1.38 (1.00-1.92)  AA= 1.78 (1.16-2.77) |
| rs7200786, A/G (*CLEC16A*) | AA: 57 (0.17)  AG: 171 (0.50)  GG: 111 (0.33) | AA: 121 (0.24)  AG: 249 (0.50)  GG: 133 (0.26) | 0.01 | 0.01 | AG= 1.21 (1.0-1.69)  GG= 1.78 (1.16-2.77) |
| rs13401, G/A (*ATF6*) | GG: 18 (0.05)  GA: 152 (0.45)  AA: 166 (0.50) | GG: 31 (0.06)  GA: 163 (0.32)  AA: 309 (0.62) | 0.0008 | 0.001 | GA= 1.73 (1.28-2.34)  GG= 1.10 (1.00-2.06) |
| rs11614913, C/T (*MIR196A2*) | CC: 146 (0.44)  CT: 139 (0.42)  TT: 45 (0.14) | CC: 182 (0.36)  CT: 229 (0.46)  TT: 92 (0.18) | 0.04 | 0.02 | CT= 1.33 (1.00-1.81)  CC= 1.63 (1.06-2.56) |
| rs755622, G/C (*MIF*) | GG: 248 (0.75)  GC: 72 (0.22)  CC: 10 (0.03) | GG: 332 (0.66)  GC: 155 (0.31)  CC: 16 (0.03) | 0.01 | 0.01 | GC= 1.61 (1.16-2.27)  GG= 1.20 (1.00-3.03) |
| rs6897932, C/T (*IL7R*) | CC:212 (0.62)  CT: 109 (0.32)  TT: 20 (0.06) | CC: 270 (0.54)  CT: 193 (0.38)  TT: 40 (0.08) | 0.04 | 0.03 | CT= 1.40 (1.03-1.72)  CC= 1.58 (1.00- 2.94) |
| rs35349669, C/T (*INPP5D*) | CC: 120 (0.36)  CT: 167 (0.49)  TT: 52 (0.15) | CC: 149 (0.30)  CT: 245 (0.49)  TT: 109 (0.21) | 0.04 | 0.02 | CT= 1.19 (1.00-1.63)  CC= 1.69 (1.11-2.63) |
| rs3745453, A/G (*ZSWIM4*) | AA: 181 (0.56)  AG: 119 (0.36)  GG: 28 (0.08) | AA: 229 (0.45)  AG: 225 (0.44)  GG: 49 (0.10) | 0.02 | 0.01 | AG= 1.51 (1.11-2.04)  AA=1.38 (1.0-2.43) |
| rs9331896, C/T (*CLU*) | CC: 44 (0.13)  CT: 140 (0.42)  TT: 152 (0.45) | CC: 78 (0.16)  CT: 242 (0.48)  TT: 183 (0.36) | 0.03 | 0.02 | CT= 1.44 (1.05-1.96)  TT= 1.49 (1.00-2.32) |
