# Supplementary material for: Immune System and Neuroinflammation in Idiopathic Parkinson’s Disease: Association Analysis of Genetic Variants and miRNAs Interactions
Source: Front Genet. 2021 Jun 3;12:651971. doi: 10.3389/fgene.2021.651971 (PMC8209518; doi:10.3389/fgene.2021.651971)
Supplement: Supplementary Table 2 — Assessment of statistical difference between male and female patients by t-test and fisher-test. Significance threshold were fixed at p < 0.05. [file Table_2.docx]

**Supplementary Table 2.** Assessment of statistical difference between male and female patients. Significance thresholds for raw and corrected *p-values* were fixed at *p-value*<0.05. UPDRS III: Unified Parkinson's Disease Rating Scale part III.

|  | **Age** | **Age of Diagnosis** | **Age of Onset** | **Disease Duration** | **UPDRS III** | **Familiarity** |
| --- | --- | --- | --- | --- | --- | --- |
| ***p-value*** | 0.155 | 0.015 | 0.058 | 0.001 | 0.823 | 0.291 |
| **Bonferroni corrected *p-value*** | 0.928 | 0.091 | 0.353 | **0.006** | 1.000 | 1.000 |
