# Supplementary material for: Immune System and Neuroinflammation in Idiopathic Parkinson’s Disease: Association Analysis of Genetic Variants and miRNAs Interactions
Source: Front Genet. 2021 Jun 3;12:651971. doi: 10.3389/fgene.2021.651971 (PMC8209518; doi:10.3389/fgene.2021.651971)
Supplement: Supplementary Table 3 — This table reports the results for the correlation tests between variants co-occurrence in the same patient and clinical data. Conf. Low, Lower Confidence Interval; Conf. High, Higher Confidence Interval. [file Table_3.docx]

**Supplementary Table 3:** This table reports the results for the correlation tests between variants co-occurrence in the same patient and clinical data. Conf. Low= Lower Confidence Interval. Conf. High= Higher Confidence Interval.

| **Pearson’s Correlation for numerical variables** | | | | | |
| --- | --- | --- | --- | --- | --- |
| **Term** | **Estimate** | **Conf. Low** | **Conf. High** | ***p-value*** | **Adjusted *p-value*** |
| **Age** | 0.023 | 0.082 | 0.129 | 0.660 | 0.699 |
| **Age of Diagnosis** | 0.114 | 0.018 | 0.244 | 0.092 | 0.230 |
| **Age of onset** | 0.076 | 0.029 | 0.181 | 0.157 | 0.263 |
| **Disease Duration** | 0.071 | 0.176 | 0.034 | 0.186 | 0.280 |
| **UPDRS** | 0.136 | 0.240 | 0.028 | 0.013 | 0.065 |
| **T-test for categorical variables** | | | | | |
| **Term** | **t** | **df** | ***p-value*** |  |  |
| **Gender** | -0.700 | 1646.4 | 0.478 |  |  |
| **Familiarity** | -0.574 | 194.02 | 0.566 |  |  |
