# Supplementary material for: Immune System and Neuroinflammation in Idiopathic Parkinson’s Disease: Association Analysis of Genetic Variants and miRNAs Interactions
Source: Front Genet. 2021 Jun 3;12:651971. doi: 10.3389/fgene.2021.651971 (PMC8209518; doi:10.3389/fgene.2021.651971)
Supplement: Supplementary Table 4 — Results concerning the significant epistatic interactions for the SNPs of interest. [file Table_4.docx]

**Supplementary Table 4. List of significant epistatic interactions for the SNPs of interest.**

| **marker1** | **marker2** | **W** | **K** | **pair.p-value** | **q.value** | **lfdr** | **bonf** |
| --- | --- | --- | --- | --- | --- | --- | --- |
| rs429358 | rs11218343 | 61,5013 | 7 | 6,43387E-11 | 2,1E-08 | 5,5E-06 | 2,09101E-08 |
| rs429358 | rs13401 | 49,0616 | 8 | 3,89248E-08 | 6,3E-06 | 1,3E-05 | 1,26505E-05 |
| rs429358 | rs1800795 | 48,6842 | 9 | 1,54281E-07 | 1,3E-05 | 3E-05 | 5,01412E-05 |
| rs429358 | rs3746444 | 45,8864 | 8 | 1,59474E-07 | 1,3E-05 | 3E-05 | 5,18291E-05 |
| rs429358 | rs24168 | 46,3918 | 9 | 4,15226E-07 | 2,7E-05 | 5,3E-05 | 0,000134948 |
| rs429358 | rs2303759 | 43,8706 | 9 | 1,22291E-06 | 6,6E-05 | 9,8E-05 | 0,000397446 |
| rs11218343 | rs190982 | 40,8125 | 8 | 1,47977E-06 | 6,9E-05 | 0,00011 | 0,000480924 |
| rs11218343 | rs1800795 | 38,359 | 7 | 2,27108E-06 | 8,2E-05 | 0,00014 | 0,000738102 |
| rs429358 | rs190982 | 41,997 | 9 | 2,71145E-06 | 8,2E-05 | 0,00016 | 0,000881222 |
| rs429358 | rs729022 | 41,9671 | 9 | 2,74598E-06 | 8,2E-05 | 0,00016 | 0,000892444 |
| rs2303759 | rs6964 | 41,9404 | 9 | 2,77726E-06 | 8,2E-05 | 0,00016 | 0,00090261 |
| rs429358 | rs2075650 | 37,1434 | 8 | 7,23732E-06 | 0,00016 | 0,00029 | 0,002352127 |
| rs429358 | rs1077667 | 37,0861 | 8 | 7,41771E-06 | 0,00016 | 0,00029 | 0,002410757 |
| rs190982 | rs13401 | 39,5341 | 9 | 7,65132E-06 | 0,00016 | 0,0003 | 0,002486679 |
| rs190982 | rs24168 | 39,4214 | 9 | 8,02102E-06 | 0,00016 | 0,00031 | 0,00260683 |
| rs3746444 | rs190982 | 39,1932 | 9 | 8,82472E-06 | 0,00016 | 0,00033 | 0,002868035 |
| rs11218343 | rs3746444 | 35,2042 | 7 | 9,02952E-06 | 0,00016 | 0,00033 | 0,002934593 |
| rs24168 | rs13401 | 38,6876 | 9 | 1,09E-05 | 0,00019 | 0,00038 | 0,003542493 |
| rs190982 | rs6964 | 38,5598 | 9 | 1,14971E-05 | 0,00019 | 0,00039 | 0,003736556 |
| rs429358 | rs12368653 | 38,3289 | 9 | 1,26583E-05 | 0,0002 | 0,00042 | 0,004113943 |
| rs1800795 | rs2303759 | 38,2068 | 9 | 1,33187E-05 | 0,0002 | 0,00043 | 0,004328565 |
| rs2303759 | rs13401 | 37,9041 | 9 | 1,51054E-05 | 0,00021 | 0,00047 | 0,004909261 |
| rs190982 | rs11614913 | 37,4651 | 9 | 1,81261E-05 | 0,00025 | 0,00053 | 0,005890968 |
| rs3746444 | rs13401 | 37,0591 | 9 | 2,14463E-05 | 0,00027 | 0,00059 | 0,006970036 |
| rs2303759 | rs7200786 | 37,0361 | 9 | 2,16511E-05 | 0,00027 | 0,0006 | 0,007036598 |
| rs429358 | rs874628 | 34,2277 | 8 | 2,51221E-05 | 0,0003 | 0,00066 | 0,008164683 |
| rs11218343 | rs13401 | 32,3805 | 7 | 3,06097E-05 | 0,00033 | 0,00076 | 0,009948142 |
| rs1800795 | rs190982 | 36,1623 | 9 | 3,10558E-05 | 0,00033 | 0,00077 | 0,010093151 |
| rs11218343 | rs2303759 | 33,6069 | 8 | 3,26721E-05 | 0,00033 | 0,00079 | 0,010618435 |
| rs1800795 | rs12368653 | 35,9779 | 9 | 3,3505E-05 | 0,00033 | 0,00081 | 0,010889127 |
| rs2303759 | rs190982 | 35,8589 | 9 | 3,51859E-05 | 0,00033 | 0,00084 | 0,011435406 |
| rs429358 | rs6964 | 35,8505 | 9 | 3,53076E-05 | 0,00033 | 0,00084 | 0,011474981 |
| rs1077667 | rs13401 | 35,8127 | 9 | 3,58599E-05 | 0,00033 | 0,00085 | 0,011654469 |
| rs3746444 | rs11614913 | 35,7661 | 9 | 3,65529E-05 | 0,00033 | 0,00086 | 0,0118797 |
| rs874628 | rs3746444 | 33,2955 | 8 | 3,72646E-05 | 0,00033 | 0,00087 | 0,012110983 |
| rs2303759 | rs3746444 | 35,6817 | 9 | 3,78432E-05 | 0,00033 | 0,00088 | 0,01229905 |
| rs729022 | rs6964 | 35,4522 | 9 | 4,15813E-05 | 0,00036 | 0,00094 | 0,013513936 |
| rs874628 | rs190982 | 35,1089 | 9 | 4,78621E-05 | 0,00039 | 0,00103 | 0,015555167 |
| rs429358 | rs7200786 | 35,0851 | 9 | 4,83305E-05 | 0,00039 | 0,00104 | 0,015707398 |
| rs729022 | rs190982 | 34,622 | 9 | 5,84027E-05 | 0,00046 | 0,00119 | 0,018980892 |
| rs729022 | rs3746444 | 34,4081 | 9 | 6,37283E-05 | 0,00049 | 0,00127 | 0,02071171 |
| rs1800795 | rs24168 | 34,2969 | 9 | 6,66818E-05 | 0,0005 | 0,00131 | 0,021671599 |
| rs3746444 | rs12368653 | 34,0837 | 9 | 7,27307E-05 | 0,00053 | 0,00139 | 0,023637486 |
| rs729022 | rs12368653 | 34,0528 | 9 | 7,36512E-05 | 0,00053 | 0,0014 | 0,023936639 |
| rs429358 | rs2546890 | 33,7637 | 9 | 8,28326E-05 | 0,00059 | 0,00153 | 0,026920586 |
| rs429358 | rs10466829 | 33,6869 | 9 | 8,54547E-05 | 0,00059 | 0,00156 | 0,027772793 |
| rs1800795 | rs13401 | 33,5743 | 9 | 8,94493E-05 | 0,00061 | 0,00161 | 0,029071018 |
| rs2303759 | rs12368653 | 33,3913 | 9 | 9,63372E-05 | 0,00064 | 0,0017 | 0,031309592 |
| rs3746444 | rs24168 | 32,5942 | 9 | 0,000132935 | 0,00086 | 0,00215 | 0,043203812 |
| rs429358 | rs6897932 | 32,2829 | 9 | 0,000150677 | 0,00095 | 0,00235 | 0,048970101 |
| rs1800795 | rs3746444 | 32,2678 | 9 | 0,000151599 | 0,00095 | 0,00236 | 0,049269722 |
| rs3746444 | rs6964 | 32,1309 | 9 | 0,000160169 | 0,00098 | 0,00246 | 0,05205505 |
| rs2303759 | rs6897932 | 32,0492 | 9 | 0,000165509 | 0,001 | 0,00252 | 0,053790404 |
| rs190982 | rs10466829 | 31,8577 | 9 | 0,000178725 | 0,00106 | 0,00267 | 0,058085686 |
| rs190982 | rs7200786 | 31,6872 | 9 | 0,000191356 | 0,00111 | 0,0028 | 0,062190714 |
| rs24168 | rs7200786 | 31,6413 | 9 | 0,000194908 | 0,00111 | 0,00284 | 0,063345115 |
| rs24168 | rs6964 | 31,5585 | 9 | 0,000201475 | 0,00111 | 0,00291 | 0,065479378 |
| rs429358 | rs3745453 | 29,2456 | 8 | 0,000201853 | 0,00111 | 0,00292 | 0,065602119 |
| rs729022 | rs7200786 | 31,3671 | 9 | 0,000217491 | 0,00118 | 0,00308 | 0,070684581 |
| rs1800795 | rs6964 | 31,2601 | 9 | 0,00022698 | 0,00121 | 0,00318 | 0,073768653 |
| rs12368653 | rs13401 | 31,1928 | 9 | 0,000233163 | 0,00122 | 0,00325 | 0,07577782 |
| rs670139 | rs6964 | 31,1627 | 9 | 0,000235974 | 0,00122 | 0,00328 | 0,076691488 |
| rs2075650 | rs13401 | 31,121 | 9 | 0,000239935 | 0,00122 | 0,00332 | 0,077979001 |
| rs7200786 | rs13401 | 30,969 | 9 | 0,000254907 | 0,00127 | 0,00347 | 0,082844865 |
| rs12368653 | rs190982 | 30,8364 | 9 | 0,000268728 | 0,00131 | 0,00361 | 0,087336617 |
| rs11218343 | rs6964 | 28,5181 | 8 | 0,000272196 | 0,00131 | 0,00365 | 0,088463653 |
| rs1800795 | rs729022 | 30,7701 | 9 | 0,000275905 | 0,00131 | 0,00369 | 0,089669274 |
| rs729022 | rs2303759 | 30,7582 | 9 | 0,00027721 | 0,00131 | 0,0037 | 0,090093333 |
| rs2283792 | rs190982 | 30,6665 | 9 | 0,000287504 | 0,00132 | 0,0038 | 0,093438924 |
| rs24168 | rs2724377 | 30,6415 | 9 | 0,000290369 | 0,00132 | 0,00383 | 0,094369902 |
| rs6964 | rs13401 | 30,6294 | 9 | 0,000291772 | 0,00132 | 0,00385 | 0,094825788 |
| rs1077667 | rs190982 | 30,5932 | 9 | 0,000295991 | 0,00132 | 0,00389 | 0,09619718 |
| rs2075650 | rs190982 | 30,5087 | 9 | 0,000306094 | 0,00134 | 0,00399 | 0,099480407 |
| rs1800795 | rs2075650 | 30,4874 | 9 | 0,000308685 | 0,00134 | 0,00401 | 0,100322498 |
| rs2303759 | rs24168 | 30,3956 | 9 | 0,000320133 | 0,00137 | 0,00413 | 0,104043139 |
| rs729022 | rs13401 | 30,1664 | 9 | 0,000350558 | 0,00147 | 0,00442 | 0,113931295 |
| rs3746444 | rs10466829 | 30,1446 | 9 | 0,000353596 | 0,00147 | 0,00445 | 0,114918717 |
| rs2075650 | rs24168 | 30,1021 | 9 | 0,000359597 | 0,00147 | 0,00451 | 0,116868921 |
| rs13401 | rs6897932 | 30,0917 | 9 | 0,000361071 | 0,00147 | 0,00452 | 0,117348115 |
| rs2303759 | rs874628 | 29,8977 | 9 | 0,000389839 | 0,00156 | 0,0048 | 0,126697795 |
| rs3746444 | rs1077667 | 29,7356 | 9 | 0,000415589 | 0,00165 | 0,00504 | 0,135066367 |
| rs874628 | rs24168 | 29,6431 | 9 | 0,000431034 | 0,00166 | 0,00519 | 0,140085988 |
| rs2075650 | rs2303759 | 29,6367 | 9 | 0,000432113 | 0,00166 | 0,0052 | 0,140436873 |
| rs24168 | rs10466829 | 29,6161 | 9 | 0,000435633 | 0,00166 | 0,00523 | 0,141580585 |
| rs190982 | rs2724377 | 29,59 | 9 | 0,000440139 | 0,00166 | 0,00527 | 0,143045137 |
| rs12368653 | rs7200786 | 29,5417 | 9 | 0,000448598 | 0,00168 | 0,00535 | 0,145794421 |
| rs190982 | rs6897932 | 29,3305 | 9 | 0,000487447 | 0,0018 | 0,00571 | 0,158420402 |
| rs11218343 | rs12368653 | 25,7046 | 7 | 0,000511579 | 0,00187 | 0,00593 | 0,166263318 |
| rs12368653 | rs24168 | 29,0609 | 9 | 0,000541859 | 0,00196 | 0,0062 | 0,176104158 |
| rs10466829 | rs35349669 | 28,8669 | 9 | 0,000584665 | 0,00207 | 0,00658 | 0,190016219 |
| rs1800795 | rs7200786 | 28,8656 | 9 | 0,000584963 | 0,00207 | 0,00658 | 0,190112863 |
| rs11218343 | rs729022 | 26,5758 | 8 | 0,000600061 | 0,0021 | 0,00672 | 0,195019953 |
| rs429358 | rs755622 | 28,7214 | 9 | 0,000618905 | 0,00211 | 0,00688 | 0,201144154 |
| rs2303759 | rs1077667 | 28,7094 | 9 | 0,000621819 | 0,00211 | 0,00691 | 0,20209125 |
| rs1800795 | rs6897932 | 28,7092 | 9 | 0,000621864 | 0,00211 | 0,00691 | 0,202105675 |
| rs2283792 | rs13401 | 28,6058 | 9 | 0,00064749 | 0,00217 | 0,00713 | 0,210434214 |
| rs2546890 | rs6964 | 28,5687 | 9 | 0,000656931 | 0,00218 | 0,00722 | 0,213502692 |
| rs2075650 | rs3746444 | 28,5282 | 9 | 0,000667417 | 0,00219 | 0,00731 | 0,216910431 |
| rs429358 | rs2283792 | 28,5031 | 9 | 0,000673977 | 0,00219 | 0,00736 | 0,219042401 |
| rs429358 | rs11614913 | 28,3724 | 9 | 0,000709208 | 0,00228 | 0,00767 | 0,230492715 |
| rs2075650 | rs12368653 | 28,3206 | 9 | 0,000723666 | 0,00228 | 0,00779 | 0,235191562 |
| rs12368653 | rs6964 | 28,3204 | 9 | 0,000723745 | 0,00228 | 0,00779 | 0,235217094 |
| rs3746444 | rs7200786 | 28,1671 | 9 | 0,000768239 | 0,0024 | 0,00817 | 0,249677687 |
| rs874628 | rs6964 | 28,1295 | 9 | 0,000779547 | 0,00241 | 0,00827 | 0,253352643 |
| rs429358 | rs9331896 | 27,9311 | 9 | 0,000842012 | 0,00258 | 0,0088 | 0,273653987 |
| rs2283792 | rs24168 | 27,8412 | 9 | 0,000871889 | 0,00265 | 0,00905 | 0,283364045 |
| rs1800795 | rs874628 | 27,796 | 9 | 0,000887314 | 0,00267 | 0,00917 | 0,288376976 |
| rs190982 | rs35349669 | 27,7232 | 9 | 0,000912706 | 0,00272 | 0,00938 | 0,296629606 |
| rs729022 | rs24168 | 27,5828 | 9 | 0,000963667 | 0,00285 | 0,0098 | 0,313191786 |
| rs729022 | rs2075650 | 27,469 | 9 | 0,001007002 | 0,00294 | 0,01016 | 0,327275724 |
| rs429358 | rs670139 | 27,431 | 9 | 0,001021907 | 0,00294 | 0,01028 | 0,332119794 |
| rs190982 | rs2546890 | 27,4306 | 9 | 0,001022074 | 0,00294 | 0,01028 | 0,332174004 |
| rs429358 | rs2724377 | 27,2791 | 9 | 0,001083598 | 0,00309 | 0,01078 | 0,352169409 |
| rs11218343 | rs24168 | 25,0074 | 8 | 0,001125849 | 0,00318 | 0,01112 | 0,365900949 |
| rs13401 | rs755622 | 27,0075 | 9 | 0,001203095 | 0,00337 | 0,01174 | 0,391005922 |
| rs874628 | rs13401 | 26,8421 | 9 | 0,001282058 | 0,00356 | 0,01236 | 0,416668976 |
| rs1800795 | rs2724377 | 26,5868 | 9 | 0,001413927 | 0,00389 | 0,0134 | 0,459526326 |
| rs1800795 | rs10466829 | 26,4078 | 9 | 0,001514189 | 0,00414 | 0,01417 | 0,492111476 |
| rs190982 | rs755622 | 26,2051 | 9 | 0,001636049 | 0,00443 | 0,01511 | 0,531715947 |
| rs24168 | rs35349669 | 26,1259 | 9 | 0,001686219 | 0,00453 | 0,01549 | 0,548021111 |
| rs12368653 | rs6897932 | 26,0997 | 9 | 0,00170309 | 0,00454 | 0,01562 | 0,553504299 |
| rs2303759 | rs2546890 | 25,9543 | 9 | 0,001800083 | 0,00476 | 0,01635 | 0,585027055 |
| rs874628 | rs12368653 | 25,9019 | 9 | 0,001836316 | 0,00476 | 0,01662 | 0,596802779 |
| rs2303759 | rs35349669 | 25,8707 | 9 | 0,001858189 | 0,00476 | 0,01679 | 0,603911554 |
| rs11218343 | rs874628 | 22,4942 | 7 | 0,00189283 | 0,00476 | 0,01705 | 0,615169609 |
| rs2303759 | rs10466829 | 25,7923 | 9 | 0,001914361 | 0,00476 | 0,01721 | 0,622167226 |
| rs2303759 | rs2283792 | 25,7824 | 9 | 0,001921564 | 0,00476 | 0,01726 | 0,624508272 |
| rs190982 | rs3745453 | 25,7719 | 9 | 0,00192925 | 0,00476 | 0,01732 | 0,627006283 |
| rs1800795 | rs1077667 | 25,7704 | 9 | 0,001930325 | 0,00476 | 0,01733 | 0,627355764 |
| rs670139 | rs13401 | 25,7552 | 9 | 0,001941498 | 0,00476 | 0,01741 | 0,630986828 |
| rs874628 | rs3745453 | 25,7498 | 9 | 0,001945492 | 0,00476 | 0,01744 | 0,632284912 |
| rs1800795 | rs755622 | 25,7228 | 9 | 0,001965552 | 0,00476 | 0,01759 | 0,638804341 |
| rs874628 | rs7200786 | 25,7191 | 9 | 0,001968307 | 0,00476 | 0,01761 | 0,63969969 |
| rs10466829 | rs13401 | 25,7057 | 9 | 0,001978361 | 0,00476 | 0,01769 | 0,642967445 |
| rs1800795 | rs11614913 | 25,5628 | 9 | 0,002088491 | 0,00498 | 0,0185 | 0,678759705 |
| rs24168 | rs9331896 | 25,5496 | 9 | 0,002098934 | 0,00498 | 0,01858 | 0,682153654 |
| rs3746444 | rs35349669 | 25,497 | 9 | 0,002141138 | 0,00504 | 0,01889 | 0,695869869 |
| rs11218343 | rs1077667 | 22,1521 | 7 | 0,002171113 | 0,00508 | 0,01911 | 0,705611663 |
| rs11218343 | rs7200786 | 23,2494 | 8 | 0,002254886 | 0,00523 | 0,01973 | 0,732837942 |
| rs6964 | rs7200786 | 25,199 | 9 | 0,002396349 | 0,00549 | 0,02077 | 0,778813295 |
| rs670139 | rs190982 | 25,1957 | 9 | 0,002399316 | 0,00549 | 0,02079 | 0,779777711 |
| rs11218343 | rs755622 | 20,5112 | 6 | 0,002497939 | 0,00568 | 0,02151 | 0,811830236 |
| rs13401 | rs11614913 | 24,9744 | 9 | 0,002607924 | 0,00589 | 0,0223 | 0,847575363 |
| rs12368653 | rs10466829 | 24,9415 | 9 | 0,002640356 | 0,00592 | 0,02254 | 0,858115849 |
| rs729022 | rs10466829 | 24,9136 | 9 | 0,002668255 | 0,00594 | 0,02274 | 0,867182902 |
| rs3746444 | rs755622 | 24,8286 | 9 | 0,002754778 | 0,00609 | 0,02336 | 0,895302801 |
| rs1800795 | rs2283792 | 24,7631 | 9 | 0,002823341 | 0,0062 | 0,02386 | 0,917585962 |
| rs3746444 | rs2724377 | 24,677 | 9 | 0,002916081 | 0,00636 | 0,02452 | 0,947726213 |
| rs2546890 | rs10466829 | 24,5813 | 9 | 0,003022536 | 0,00655 | 0,02529 | 0,982324179 |
| rs670139 | rs2303759 | 24,4858 | 9 | 0,003132494 | 0,00674 | 0,02607 | 1 |
| rs3746444 | rs6897932 | 24,4515 | 9 | 0,003172828 | 0,00676 | 0,02636 | 1 |
| rs2303759 | rs755622 | 24,4447 | 9 | 0,003180942 | 0,00676 | 0,02641 | 1 |
| rs2303759 | rs2724377 | 24,3859 | 9 | 0,003251675 | 0,00685 | 0,02692 | 1 |
| rs10466829 | rs6964 | 24,3723 | 9 | 0,003268116 | 0,00685 | 0,02703 | 1 |
| rs670139 | rs3746444 | 24,1768 | 9 | 0,003515354 | 0,00732 | 0,02879 | 1 |
| rs3746444 | rs2283792 | 23,9242 | 9 | 0,003861752 | 0,00788 | 0,03123 | 1 |
| rs1800795 | rs3745453 | 23,9142 | 9 | 0,003876086 | 0,00788 | 0,03133 | 1 |
| rs1077667 | rs12368653 | 23,9107 | 9 | 0,003881141 | 0,00788 | 0,03137 | 1 |
| rs24168 | rs6897932 | 23,9106 | 9 | 0,003881243 | 0,00788 | 0,03137 | 1 |
| rs11218343 | rs6897932 | 21,7722 | 8 | 0,004001696 | 0,00804 | 0,03221 | 1 |
| rs729022 | rs6897932 | 23,8237 | 9 | 0,004008419 | 0,00804 | 0,03226 | 1 |
| rs429358 | rs35349669 | 23,5736 | 9 | 0,004397269 | 0,00872 | 0,03499 | 1 |
| rs3746444 | rs2546890 | 23,5726 | 9 | 0,004398927 | 0,00872 | 0,035 | 1 |
| rs1077667 | rs24168 | 23,4525 | 9 | 0,004598409 | 0,00897 | 0,0364 | 1 |
| rs3746444 | rs3745453 | 23,4457 | 9 | 0,004610092 | 0,00897 | 0,03648 | 1 |
| rs729022 | rs874628 | 23,4213 | 9 | 0,004651794 | 0,00897 | 0,03677 | 1 |
| rs1800795 | rs35349669 | 23,4174 | 9 | 0,004658464 | 0,00897 | 0,03682 | 1 |
| rs11218343 | rs10466829 | 21,3719 | 8 | 0,004666567 | 0,00897 | 0,03687 | 1 |
| rs1077667 | rs6964 | 23,3509 | 9 | 0,004774094 | 0,00913 | 0,03763 | 1 |
| rs1800795 | rs2546890 | 22,917 | 9 | 0,005599108 | 0,01064 | 0,04339 | 1 |
| rs2075650 | rs1077667 | 20,8399 | 8 | 0,00571735 | 0,01075 | 0,04422 | 1 |
| rs2724377 | rs13401 | 22,8566 | 9 | 0,005724294 | 0,01075 | 0,04427 | 1 |
| rs6964 | rs755622 | 22,8088 | 9 | 0,005825288 | 0,01077 | 0,04497 | 1 |
| rs6964 | rs6897932 | 22,8076 | 9 | 0,005828021 | 0,01077 | 0,04499 | 1 |
| rs729022 | rs1077667 | 22,8055 | 9 | 0,005832393 | 0,01077 | 0,04502 | 1 |
| rs2546890 | rs13401 | 22,7792 | 9 | 0,005888744 | 0,01081 | 0,04542 | 1 |
| rs190982 | rs9331896 | 22,671 | 9 | 0,006126106 | 0,01119 | 0,04708 | 1 |
| rs13401 | rs3745453 | 22,6192 | 9 | 0,006243013 | 0,01134 | 0,0479 | 1 |
| rs24168 | rs755622 | 22,5692 | 9 | 0,006357854 | 0,01148 | 0,04871 | 1 |
| rs2075650 | rs6964 | 22,5482 | 9 | 0,006406818 | 0,0115 | 0,04905 | 1 |
| rs2303759 | rs11614913 | 22,3927 | 9 | 0,006779885 | 0,01211 | 0,05167 | 1 |
| rs2303759 | rs3745453 | 22,3146 | 9 | 0,006974963 | 0,01239 | 0,05305 | 1 |
| rs729022 | rs755622 | 22,2628 | 9 | 0,007107282 | 0,01255 | 0,05398 | 1 |
| rs10466829 | rs9331896 | 22,2174 | 9 | 0,007225449 | 0,01259 | 0,05481 | 1 |
| rs2075650 | rs7200786 | 22,2138 | 9 | 0,007234859 | 0,01259 | 0,05488 | 1 |
| rs24168 | rs11614913 | 22,2106 | 9 | 0,00724317 | 0,01259 | 0,05494 | 1 |
| rs2283792 | rs10466829 | 22,158 | 9 | 0,007382718 | 0,01276 | 0,05593 | 1 |
| rs13401 | rs35349669 | 22,1375 | 9 | 0,007437735 | 0,01279 | 0,05632 | 1 |
| rs670139 | rs24168 | 22,099 | 9 | 0,007542173 | 0,01287 | 0,05706 | 1 |
| rs1077667 | rs2283792 | 22,0913 | 9 | 0,007563041 | 0,01287 | 0,0572 | 1 |
| rs2075650 | rs874628 | 20,0535 | 8 | 0,007698932 | 0,01303 | 0,05817 | 1 |
| rs2283792 | rs6964 | 21,9984 | 9 | 0,007821433 | 0,01308 | 0,05904 | 1 |
| rs2546890 | rs24168 | 21,99 | 9 | 0,007845362 | 0,01308 | 0,05921 | 1 |
| rs1800795 | rs670139 | 21,9892 | 9 | 0,007847521 | 0,01308 | 0,05922 | 1 |
| rs24168 | rs3745453 | 21,7924 | 9 | 0,008425069 | 0,0139 | 0,06334 | 1 |
| rs12368653 | rs2546890 | 21,792 | 9 | 0,008426178 | 0,0139 | 0,06335 | 1 |
| rs2303759 | rs9331896 | 21,6998 | 9 | 0,008710682 | 0,01429 | 0,06539 | 1 |
| rs874628 | rs6897932 | 21,6864 | 9 | 0,008752654 | 0,01429 | 0,06569 | 1 |
| rs874628 | rs2546890 | 21,5402 | 9 | 0,009224586 | 0,01494 | 0,06909 | 1 |
| rs6964 | rs11614913 | 21,5277 | 9 | 0,009265972 | 0,01494 | 0,06939 | 1 |
| rs729022 | rs2724377 | 21,5219 | 9 | 0,00928532 | 0,01494 | 0,06953 | 1 |
| rs2283792 | rs3745453 | 21,4831 | 9 | 0,009415573 | 0,01507 | 0,07047 | 1 |
| rs874628 | rs1077667 | 21,3979 | 9 | 0,009707422 | 0,01547 | 0,07259 | 1 |
| rs6964 | rs35349669 | 21,384 | 9 | 0,009755582 | 0,01547 | 0,07294 | 1 |
| rs670139 | rs1077667 | 21,3417 | 9 | 0,00990453 | 0,01563 | 0,07402 | 1 |
| rs6964 | rs3745453 | 21,2302 | 9 | 0,010306961 | 0,01618 | 0,07697 | 1 |
| rs7200786 | rs11614913 | 21,2004 | 9 | 0,01041735 | 0,01628 | 0,07777 | 1 |
| rs2075650 | rs2546890 | 21,183 | 9 | 0,010482134 | 0,0163 | 0,07825 | 1 |
| rs874628 | rs2283792 | 21,0674 | 9 | 0,010922721 | 0,01675 | 0,0815 | 1 |
| rs6964 | rs2724377 | 21,0632 | 9 | 0,010939381 | 0,01675 | 0,08162 | 1 |
| rs1077667 | rs2724377 | 21,0535 | 9 | 0,010977035 | 0,01675 | 0,0819 | 1 |
| rs11218343 | rs11614913 | 18,0031 | 7 | 0,010990197 | 0,01675 | 0,082 | 1 |
| rs3746444 | rs9331896 | 21,0402 | 9 | 0,011029224 | 0,01675 | 0,08229 | 1 |
| rs12368653 | rs3745453 | 20,8648 | 9 | 0,011738367 | 0,01774 | 0,08756 | 1 |
| rs10466829 | rs7200786 | 20,7628 | 9 | 0,012170667 | 0,01831 | 0,09081 | 1 |
| rs874628 | rs10466829 | 20,619 | 9 | 0,012805591 | 0,01915 | 0,09561 | 1 |
| rs2283792 | rs12368653 | 20,6108 | 9 | 0,012842696 | 0,01915 | 0,09589 | 1 |
| rs1077667 | rs7200786 | 20,445 | 9 | 0,013616296 | 0,02018 | 0,1018 | 1 |
| rs12368653 | rs2724377 | 20,4363 | 9 | 0,013657861 | 0,02018 | 0,10212 | 1 |
| rs729022 | rs2283792 | 20,4165 | 9 | 0,013753397 | 0,02023 | 0,10286 | 1 |
| rs6964 | rs9331896 | 20,3413 | 9 | 0,014122326 | 0,02067 | 0,10571 | 1 |
| rs670139 | rs11614913 | 20,2594 | 9 | 0,01453466 | 0,02118 | 0,10891 | 1 |
| rs729022 | rs35349669 | 20,2159 | 9 | 0,014758149 | 0,02135 | 0,11066 | 1 |
| rs11218343 | rs670139 | 17,2188 | 7 | 0,014781306 | 0,02135 | 0,11084 | 1 |
| rs874628 | rs35349669 | 20,18 | 9 | 0,014944974 | 0,02149 | 0,11212 | 1 |
| rs13401 | rs9331896 | 20,1402 | 9 | 0,015154742 | 0,0217 | 0,11377 | 1 |
| rs2283792 | rs11614913 | 20,0809 | 9 | 0,015472512 | 0,02206 | 0,11627 | 1 |
| rs670139 | rs12368653 | 19,8923 | 9 | 0,016526078 | 0,02344 | 0,12467 | 1 |
| rs1800795 | rs9331896 | 19,8815 | 9 | 0,016588769 | 0,02344 | 0,12517 | 1 |
| rs1077667 | rs6897932 | 19,811 | 9 | 0,017001089 | 0,02388 | 0,12849 | 1 |
| rs729022 | rs3745453 | 19,8035 | 9 | 0,017045431 | 0,02388 | 0,12885 | 1 |
| rs874628 | rs11614913 | 19,6115 | 9 | 0,018221402 | 0,02542 | 0,13845 | 1 |
| rs7200786 | rs755622 | 19,5808 | 9 | 0,018416272 | 0,02549 | 0,14006 | 1 |
| rs1077667 | rs11614913 | 19,5789 | 9 | 0,018428383 | 0,02549 | 0,14016 | 1 |
| rs729022 | rs2546890 | 19,3181 | 9 | 0,020166878 | 0,02777 | 0,15469 | 1 |
| rs11218343 | rs2283792 | 17,3918 | 8 | 0,020527249 | 0,02815 | 0,15775 | 1 |
| rs7200786 | rs6897932 | 19,2355 | 9 | 0,020748451 | 0,02833 | 0,15964 | 1 |
| rs2075650 | rs10466829 | 19,2218 | 9 | 0,020846357 | 0,02835 | 0,16047 | 1 |
| rs2724377 | rs7200786 | 19,1829 | 9 | 0,021127356 | 0,0286 | 0,16288 | 1 |
| rs670139 | rs2724377 | 19,1723 | 9 | 0,021204586 | 0,0286 | 0,16354 | 1 |
| rs10466829 | rs2724377 | 19,0843 | 9 | 0,021854774 | 0,02924 | 0,16915 | 1 |
| rs11218343 | rs2546890 | 16,1666 | 7 | 0,021864135 | 0,02924 | 0,16923 | 1 |
| rs874628 | rs755622 | 19,039 | 9 | 0,022197296 | 0,02957 | 0,17213 | 1 |
| rs874628 | rs2724377 | 18,9472 | 9 | 0,022906054 | 0,03039 | 0,17833 | 1 |
| rs12368653 | rs755622 | 18,615 | 9 | 0,025653187 | 0,03379 | 0,20292 | 1 |
| rs2724377 | rs35349669 | 18,6119 | 9 | 0,025679962 | 0,03379 | 0,20317 | 1 |
| rs670139 | rs7200786 | 18,5995 | 9 | 0,025788736 | 0,0338 | 0,20416 | 1 |
| rs2075650 | rs11614913 | 18,5246 | 9 | 0,026452463 | 0,03443 | 0,21025 | 1 |
| rs12368653 | rs9331896 | 18,5214 | 9 | 0,026481115 | 0,03443 | 0,21051 | 1 |
| rs2075650 | rs670139 | 18,4495 | 9 | 0,027133688 | 0,03513 | 0,21655 | 1 |
| rs12368653 | rs11614913 | 18,4333 | 9 | 0,02728346 | 0,03519 | 0,21794 | 1 |
| rs670139 | rs874628 | 18,3824 | 9 | 0,027756602 | 0,03566 | 0,22235 | 1 |
| rs10466829 | rs755622 | 18,3168 | 9 | 0,028378348 | 0,03631 | 0,2282 | 1 |
| rs12368653 | rs35349669 | 18,3022 | 9 | 0,028519355 | 0,03635 | 0,22953 | 1 |
| rs2283792 | rs7200786 | 18,2846 | 9 | 0,028688857 | 0,03642 | 0,23113 | 1 |
| rs10466829 | rs3745453 | 18,121 | 9 | 0,030312531 | 0,03833 | 0,24665 | 1 |
| rs1077667 | rs10466829 | 18,0752 | 9 | 0,030782757 | 0,03878 | 0,2512 | 1 |
| rs2546890 | rs7200786 | 18,0239 | 9 | 0,031317545 | 0,0393 | 0,2564 | 1 |
| rs6897932 | rs35349669 | 17,821 | 9 | 0,03351589 | 0,04189 | 0,27812 | 1 |
| rs11218343 | rs35349669 | 15,9597 | 8 | 0,034116309 | 0,04244 | 0,28415 | 1 |
| rs729022 | rs11614913 | 17,759 | 9 | 0,03421615 | 0,04244 | 0,28515 | 1 |
| rs2075650 | rs755622 | 15,87 | 8 | 0,035200832 | 0,04346 | 0,29513 | 1 |
| rs2075650 | rs3745453 | 15,8617 | 8 | 0,035303437 | 0,04346 | 0,29617 | 1 |
| rs11218343 | rs2724377 | 15,7085 | 8 | 0,037234798 | 0,04567 | 0,31606 | 1 |
| rs2283792 | rs6897932 | 17,4174 | 9 | 0,038322417 | 0,04682 | 0,32743 | 1 |
| rs2546890 | rs6897932 | 17,3825 | 9 | 0,038766083 | 0,04719 | 0,3321 | 1 |
| rs2546890 | rs755622 | 17,1764 | 9 | 0,041489125 | 0,05031 | 0,36121 | 1 |
| rs2546890 | rs3745453 | 17,1225 | 9 | 0,042228832 | 0,05102 | 0,36925 | 1 |
| rs2075650 | rs6897932 | 17,0988 | 9 | 0,042557825 | 0,05106 | 0,37284 | 1 |
| rs10466829 | rs6897932 | 17,0973 | 9 | 0,042578866 | 0,05106 | 0,37307 | 1 |
| rs11218343 | rs9331896 | 14,2175 | 7 | 0,044209076 | 0,05282 | 0,39102 | 1 |
| rs670139 | rs6897932 | 16,968 | 9 | 0,044418758 | 0,05288 | 0,39334 | 1 |
| rs729022 | rs9331896 | 16,917 | 9 | 0,045165262 | 0,05357 | 0,40166 | 1 |
| rs2075650 | rs2283792 | 16,6716 | 9 | 0,048916087 | 0,05781 | 0,44424 | 1 |
| rs35349669 | rs3745453 | 16,4209 | 9 | 0,053040301 | 0,06246 | 0,4925 | 1 |
| rs670139 | rs2283792 | 16,3526 | 9 | 0,054218552 | 0,06361 | 0,50655 | 1 |
| rs2075650 | rs9331896 | 16,1979 | 9 | 0,056973797 | 0,06624 | 0,53984 | 1 |
| rs874628 | rs9331896 | 16,1955 | 9 | 0,057016805 | 0,06624 | 0,54037 | 1 |
| rs7200786 | rs35349669 | 16,1926 | 9 | 0,057071184 | 0,06624 | 0,54103 | 1 |
| rs11218343 | rs3745453 | 14,4034 | 8 | 0,058156799 | 0,06726 | 0,55433 | 1 |
| rs7200786 | rs9331896 | 16,0712 | 9 | 0,059324835 | 0,06837 | 0,56873 | 1 |
| rs2075650 | rs2724377 | 15,9981 | 9 | 0,060721857 | 0,06973 | 0,58609 | 1 |
| rs7200786 | rs3745453 | 15,8069 | 9 | 0,064514719 | 0,07383 | 0,63396 | 1 |
| rs2283792 | rs9331896 | 15,5384 | 9 | 0,070205608 | 0,08006 | 0,70766 | 1 |
| rs670139 | rs2546890 | 15,3518 | 9 | 0,0744214 | 0,08457 | 0,76362 | 1 |
| rs729022 | rs670139 | 14,8504 | 9 | 0,086895552 | 0,0984 | 0,9353 | 1 |
| rs2283792 | rs2546890 | 14,7913 | 9 | 0,088480805 | 0,09985 | 0,95773 | 1 |
| rs10466829 | rs11614913 | 14,7011 | 9 | 0,090952125 | 0,10228 | 0,99294 | 1 |
| rs6897932 | rs3745453 | 14,5837 | 9 | 0,094257876 | 0,10563 | 1 | 1 |
| rs1077667 | rs2546890 | 14,5109 | 9 | 0,096363043 | 0,10762 | 1 | 1 |
| rs2546890 | rs9331896 | 14,4427 | 9 | 0,098369365 | 0,10949 | 1 | 1 |
| rs2546890 | rs35349669 | 14,3429 | 9 | 0,101375223 | 0,11245 | 1 | 1 |
| rs35349669 | rs9331896 | 14,2948 | 9 | 0,102851536 | 0,1137 | 1 | 1 |
| rs2075650 | rs35349669 | 14,0631 | 9 | 0,110230555 | 0,12144 | 1 | 1 |
| rs755622 | rs6897932 | 14,0092 | 9 | 0,112013333 | 0,12299 | 1 | 1 |
| rs2283792 | rs755622 | 13,9957 | 9 | 0,112462394 | 0,12306 | 1 | 1 |
| rs755622 | rs35349669 | 13,9272 | 9 | 0,114770984 | 0,12517 | 1 | 1 |
| rs670139 | rs10466829 | 13,7287 | 9 | 0,121689807 | 0,13227 | 1 | 1 |
| rs11614913 | rs6897932 | 13,6496 | 9 | 0,124547908 | 0,13493 | 1 | 1 |
| rs1077667 | rs755622 | 13,3935 | 9 | 0,134196169 | 0,1449 | 1 | 1 |
| rs2724377 | rs755622 | 13,2494 | 9 | 0,139899521 | 0,15055 | 1 | 1 |
| rs2724377 | rs11614913 | 13,1977 | 9 | 0,141996604 | 0,15231 | 1 | 1 |
| rs2546890 | rs2724377 | 13,1424 | 9 | 0,144265746 | 0,15423 | 1 | 1 |
| rs2724377 | rs6897932 | 12,8733 | 9 | 0,155761122 | 0,16597 | 1 | 1 |
| rs11614913 | rs9331896 | 12,8172 | 9 | 0,158253957 | 0,16808 | 1 | 1 |
| rs1077667 | rs3745453 | 12,7057 | 9 | 0,163300864 | 0,17288 | 1 | 1 |
| rs2724377 | rs9331896 | 12,3634 | 9 | 0,179642018 | 0,18936 | 1 | 1 |
| rs2283792 | rs2724377 | 12,3553 | 9 | 0,180041303 | 0,18936 | 1 | 1 |
| rs2546890 | rs11614913 | 11,9282 | 9 | 0,202318595 | 0,21211 | 1 | 1 |
| rs670139 | rs3745453 | 11,8175 | 9 | 0,208438355 | 0,21782 | 1 | 1 |
| rs2283792 | rs35349669 | 11,7806 | 9 | 0,21051363 | 0,21929 | 1 | 1 |
| rs11614913 | rs35349669 | 11,6269 | 9 | 0,21932418 | 0,22773 | 1 | 1 |
| rs1077667 | rs35349669 | 11,4867 | 9 | 0,227607742 | 0,23558 | 1 | 1 |
| rs755622 | rs9331896 | 11,3617 | 9 | 0,235199842 | 0,24267 | 1 | 1 |
| rs670139 | rs35349669 | 10,9466 | 9 | 0,261809813 | 0,26927 | 1 | 1 |
| rs670139 | rs755622 | 10,4033 | 9 | 0,299953873 | 0,30752 | 1 | 1 |
| rs2724377 | rs3745453 | 10,206 | 9 | 0,314739931 | 0,32134 | 1 | 1 |
| rs670139 | rs9331896 | 10,1972 | 9 | 0,315411157 | 0,32134 | 1 | 1 |
| rs1077667 | rs9331896 | 10,1121 | 9 | 0,321954595 | 0,32699 | 1 | 1 |
| rs6897932 | rs9331896 | 10,0933 | 9 | 0,323412361 | 0,32744 | 1 | 1 |
| rs11614913 | rs3745453 | 10,0279 | 9 | 0,328517096 | 0,33158 | 1 | 1 |
| rs11614913 | rs755622 | 9,86799 | 9 | 0,341232793 | 0,34335 | 1 | 1 |
| rs755622 | rs3745453 | 9,47624 | 9 | 0,373734498 | 0,37489 | 1 | 1 |
| rs3745453 | rs9331896 | 7,8157 | 9 | 0,530527904 | 0,53053 | 1 | 1 |
